# Supplementary material for: School-based interventions to prevent anxiety and depression in children and adolescents in low- and middle-income countries: A systematic review
Source: PLoS One. 2025 Apr 30;20(4):e0316825. doi: 10.1371/journal.pone.0316825 (PMC12043155; doi:10.1371/journal.pone.0316825)
Supplement: S1 File — (DOCX) [file pone.0316825.s001.docx]

**Supplementary file 1**

## Search strategies for each database

Ovid MEDLINE(R) ALL <1946 to July 06, 2023>

1 CHILD, PRESCHOOL/ or CHILD/ or ADOLESCENT/ or YOUNG ADULT/ 3817520

2 (child* or boy* or girl* or kids or juvenil* or minors or paediatric* or pediatric* or adolesc* or preadolesc* or preadolesc* or pubert* or pubescen* or prepube* or prepube* or teen* or (young adj (adult* or people or patient* or men* or women* or male or female or survivor* or offender* or minorit*)) or youth* or student* or undergrad*).mp. [mp=title, book title, abstract, original title, name of substance word, subject heading word, floating sub-heading word, keyword heading word, organism supplementary concept word, protocol supplementary concept word, rare disease supplementary concept word, unique identifier, synonyms, population supplementary concept word, anatomy supplementary concept word] 4976340

3 1 or 2 4976340

4 EDUCATION/ 21522

5 SCHOOLS/ or SCHOOLS, NURSERY/ 52708

6 SCHOOL HEALTH SERVICES/ or SCHOOL NURSING/ 22734

7 STUDENTS/ or UNIVERSITIES/ 109989

8 (preschool or nursery or kindergarten or school* or college* or campus* or classroom* or curricul* or teacher or gatekeeper or pupil*).mp. [mp=title, book title, abstract, original title, name of substance word, subject heading word, floating sub-heading word, keyword heading word, organism supplementary concept word, protocol supplementary concept word, rare disease supplementary concept word, unique identifier, synonyms, population supplementary concept word, anatomy supplementary concept word] 1618157

9 PEER GROUP/ 24128

10 ((peer or peers) adj (education or group or relation* or support* or intervention* or leader*)).mp. [mp=title, book title, abstract, original title, name of substance word, subject heading word, floating sub-heading word, keyword heading word, organism supplementary concept word, protocol supplementary concept word, rare disease supplementary concept word, unique identifier, synonyms, population supplementary concept word, anatomy supplementary concept word] 33867

11 student* union.mp. 39

12 ((primary or secondary or tertiary) adj educat*).mp. [mp=title, book title, abstract, original title, name of substance word, subject heading word, floating sub-heading word, keyword heading word, organism supplementary concept word, protocol supplementary concept word, rare disease supplementary concept word, unique identifier, synonyms, population supplementary concept word, anatomy supplementary concept word] 8308

13 ((church or communit* or holiday* or religi* or spiritual* or youth or vacation) adj2 (camp or club or group)).mp. [mp=title, book title, abstract, original title, name of substance word, subject heading word, floating sub-heading word, keyword heading word, organism supplementary concept word, protocol supplementary concept word, rare disease supplementary concept word, unique identifier, synonyms, population supplementary concept word, anatomy supplementary concept word] 4348

14 ((church or communit* or holiday* or religi* or spiritual* or youth or vacation) adj based).mp. [mp=title, book title, abstract, original title, name of substance word, subject heading word, floating sub-heading word, keyword heading word, organism supplementary concept word, protocol supplementary concept word, rare disease supplementary concept word, unique identifier, synonyms, population supplementary concept word, anatomy supplementary concept word] 81654

15 (university or universities).mp. [mp=title, book title, abstract, original title, name of substance word, subject heading word, floating sub-heading word, keyword heading word, organism supplementary concept word, protocol supplementary concept word, rare disease supplementary concept word, unique identifier, synonyms, population supplementary concept word, anatomy supplementary concept word] 493770

16 ((detention or refugee*) adj (camp* or centre* or center*)).mp. [mp=title, book title, abstract, original title, name of substance word, subject heading word, floating sub-heading word, keyword heading word, organism supplementary concept word, protocol supplementary concept word, rare disease supplementary concept word, unique identifier, synonyms, population supplementary concept word, anatomy supplementary concept word] 2405

17 4 or 5 or 6 or 7 or 8 or 9 or 10 or 11 or 12 or 13 or 14 or 15 or 16 2122320

18 ADAPTATION, PSYCHOLOGICAL/ 104233

19 EMOTIONS/ 84866

20 MENTAL HEALTH/ 61458

21 SOCIAL ADJUSTMENT/ 23668

22 exp STRESS, PSYCHOLOGICAL/ 152739

23 (mental health or mental* ill* or psychiatric).mp. [mp=title, book title, abstract, original title, name of substance word, subject heading word, floating sub-heading word, keyword heading word, organism supplementary concept word, protocol supplementary concept word, rare disease supplementary concept word, unique identifier, synonyms, population supplementary concept word, anatomy supplementary concept word] 559530

24 (wellbeing or well being).mp. [mp=title, book title, abstract, original title, name of substance word, subject heading word, floating sub-heading word, keyword heading word, organism supplementary concept word, protocol supplementary concept word, rare disease supplementary concept word, unique identifier, synonyms, population supplementary concept word, anatomy supplementary concept word] 140419

25 (stress* or distress*).mp. [mp=title, book title, abstract, original title, name of substance word, subject heading word, floating sub-heading word, keyword heading word, organism supplementary concept word, protocol supplementary concept word, rare disease supplementary concept word, unique identifier, synonyms, population supplementary concept word, anatomy supplementary concept word] 1402635

26 18 or 19 or 20 or 21 or 22 or 23 or 24 or 25 2095243

27 DEPRESSION/ 150744

28 Depressive Disorder/ 75020

29 Mood Disorders/ 15918

30 (depress* or dysthymi* or affective disorder* or affective symptom* or mood* or mental).mp. [mp=title, book title, abstract, original title, name of substance word, subject heading word, floating sub-heading word, keyword heading word, organism supplementary concept word, protocol supplementary concept word, rare disease supplementary concept word, unique identifier, synonyms, population supplementary concept word, anatomy supplementary concept word] 1257449

31 ((Axis 1 or Axis I) adj disorder*).mp. [mp=title, book title, abstract, original title, name of substance word, subject heading word, floating sub-heading word, keyword heading word, organism supplementary concept word, protocol supplementary concept word, rare disease supplementary concept word, unique identifier, synonyms, population supplementary concept word, anatomy supplementary concept word] 2010

32 27 or 28 or 29 or 30 or 31 1257778

33 exp ANXIETY DISORDERS/ 90406

34 ANXIETY/ 106663

35 anxi*.mp. [mp=title, book title, abstract, original title, name of substance word, subject heading word, floating sub-heading word, keyword heading word, organism supplementary concept word, protocol supplementary concept word, rare disease supplementary concept word, unique identifier, synonyms, population supplementary concept word, anatomy supplementary concept word] 324375

36 (phobi* or agoraphobi* or PTSD or post trauma* or posttrauma or panic* or OCD or obsess* or compulsi* or GAD or stress disorder* or stress reaction* or acute stress or neurosis or neuroses or neurotic or psychoneuro* or (school adj2 (refusal or avoid*)) or social avoidance or mutism).mp. [mp=title, book title, abstract, original title, name of substance word, subject heading word, floating sub-heading word, keyword heading word, organism supplementary concept word, protocol supplementary concept word, rare disease supplementary concept word, unique identifier, synonyms, population supplementary concept word, anatomy supplementary concept word] 214287

37 (((anxi* or fear or fright) adj3 (perform* or athlet* or music* or act* or test* or exam*)) or math* anxiety).mp. [mp=title, book title, abstract, original title, name of substance word, subject heading word, floating sub-heading word, keyword heading word, organism supplementary concept word, protocol supplementary concept word, rare disease supplementary concept word, unique identifier, synonyms, population supplementary concept word, anatomy supplementary concept word] 22831

38 (public adj3 (speak* or speech)).mp. [mp=title, book title, abstract, original title, name of substance word, subject heading word, floating sub-heading word, keyword heading word, organism supplementary concept word, protocol supplementary concept word, rare disease supplementary concept word, unique identifier, synonyms, population supplementary concept word, anatomy supplementary concept word] 1244

39 33 or 34 or 35 or 36 or 37 or 38 491771

40 CONDUCT DISORDER/ 3871

41 CHILD BEHAVIOR DISORDERS/ 20836

42 JUVENILE DELINQUENCY/ 9017

43 SOCIAL BEHAVIOR/ 57975

44 SOCIAL BEHAVIOR DISORDERS/ 4803

45 ((behavi* or conduct or personalit*) adj2 (agressi* or nonagressi* or antisocial or anti social or dyssocial or defiant or delinquen* or disturb* or disrupt* or disorder* or internali#ing or externali#ing or problem*)).mp. [mp=title, book title, abstract, original title, name of substance word, subject heading word, floating sub-heading word, keyword heading word, organism supplementary concept word, protocol supplementary concept word, rare disease supplementary concept word, unique identifier, synonyms, population supplementary concept word, anatomy supplementary concept word] 135512

46 ((conduct or behavi* or antisocial or anti social or dyssocial or emotional* or internali#ing or externali#ing) adj3 (problem* of difficult* or psychopathol*)).mp. [mp=title, book title, abstract, original title, name of substance word, subject heading word, floating sub-heading word, keyword heading word, organism supplementary concept word, protocol supplementary concept word, rare disease supplementary concept word, unique identifier, synonyms, population supplementary concept word, anatomy supplementary concept word] 2641

47 (oppositional adj3 (defiant* or disorder*)).mp. [mp=title, book title, abstract, original title, name of substance word, subject heading word, floating sub-heading word, keyword heading word, organism supplementary concept word, protocol supplementary concept word, rare disease supplementary concept word, unique identifier, synonyms, population supplementary concept word, anatomy supplementary concept word] 2949

48 40 or 41 or 42 or 43 or 44 or 45 or 46 or 47 197542

49 26 or 32 or 39 or 48 3049859

50 PREVENTIVE HEALTH SERVICES/ or "EARLY INTERVENTION (education)"/ or HEALTH LITERACY/ or PATIENT EDUCATION AS TOPIC/ or HEALTH PROMOTION/ or PRIMARY PREVENTION/ or SECONDARY PREVENTION/ 227634

51 prevent*.mp. [mp=title, book title, abstract, original title, name of substance word, subject heading word, floating sub-heading word, keyword heading word, organism supplementary concept word, protocol supplementary concept word, rare disease supplementary concept word, unique identifier, synonyms, population supplementary concept word, anatomy supplementary concept word] 2813185

52 ((early or brief) adj intervention*).mp. [mp=title, book title, abstract, original title, name of substance word, subject heading word, floating sub-heading word, keyword heading word, organism supplementary concept word, protocol supplementary concept word, rare disease supplementary concept word, unique identifier, synonyms, population supplementary concept word, anatomy supplementary concept word] 34342

53 ((universal or indicated or targeted) adj2 (program* or intervention*)).mp. [mp=title, book title, abstract, original title, name of substance word, subject heading word, floating sub-heading word, keyword heading word, organism supplementary concept word, protocol supplementary concept word, rare disease supplementary concept word, unique identifier, synonyms, population supplementary concept word, anatomy supplementary concept word] 27848

54 (vulnerabl* or at risk or (risk adj2 reduc*)).mp. [mp=title, book title, abstract, original title, name of substance word, subject heading word, floating sub-heading word, keyword heading word, organism supplementary concept word, protocol supplementary concept word, rare disease supplementary concept word, unique identifier, synonyms, population supplementary concept word, anatomy supplementary concept word] 503116

55 behavio* activation.mp. [mp=title, book title, abstract, original title, name of substance word, subject heading word, floating sub-heading word, keyword heading word, organism supplementary concept word, protocol supplementary concept word, rare disease supplementary concept word, unique identifier, synonyms, population supplementary concept word, anatomy supplementary concept word] 2464

56 (behavio* adj2 (intervention* or therap* or treatment* or psychotherap* or psychotherap*)).mp. [mp=title, book title, abstract, original title, name of substance word, subject heading word, floating sub-heading word, keyword heading word, organism supplementary concept word, protocol supplementary concept word, rare disease supplementary concept word, unique identifier, synonyms, population supplementary concept word, anatomy supplementary concept word] 97432

57 Behavior Therapy/ 30294

58 Cognitive Therapy/ 30264

59 self monitor*.mp. [mp=title, book title, abstract, original title, name of substance word, subject heading word, floating sub-heading word, keyword heading word, organism supplementary concept word, protocol supplementary concept word, rare disease supplementary concept word, unique identifier, synonyms, population supplementary concept word, anatomy supplementary concept word] 18089

60 (activit* adj3 (schedul* or plan* or arrang* or organis* or organiz*)).mp. [mp=title, book title, abstract, original title, name of substance word, subject heading word, floating sub-heading word, keyword heading word, organism supplementary concept word, protocol supplementary concept word, rare disease supplementary concept word, unique identifier, synonyms, population supplementary concept word, anatomy supplementary concept word] 26474

61 50 or 51 or 52 or 53 or 54 or 55 or 56 or 57 or 58 or 59 or 60 3416057

62 RISK/ or RISK FACTORS/ 1079017

63 exp ACCIDENTS/ 209054

64 BEREAVEMENT/ or GRIEF/ 15186

65 SOCIAL PROBLEMS/ 8410

66 BULLYING/ 6395

67 CHILD OF IMPAIRED PARENTS/ 5649

68 CHILD, ORPHANED/ 776

69 CRIME VICTIMS/ 13280

70 exp DISASTERS/ 99968

71 DIVORCE/ 4965

72 LIFE CHANGE EVENTS/ 23667

73 RUNAWAY BEHAVIOR/ 314

74 URBAN POPULATION/ 62752

75 RURAL POPULATION/ 69228

76 SURVIVORS/ 30406

77 VIOLENCE/ 34976

78 WARFARE/ 23151

79 social problems/ or exp civil disorders/ or exp crime/ or exp human rights abuses/ or exp parental death/ or poverty/ or exp social behavior disorders/ or domestic violence/ or exp child abuse/ or exp ethnic violence/ or physical abuse/ or exp terrorism/ or torture/ or exposure to violence/ or exp "warfare and armed conflicts"/ 249701

80 "dissent and disputes"/ or family conflict/ or psychosocial deprivation/ 10427

81 (bereave* or bullying or divorce or foster care or grief or humanitarian or orphan* or RTA or refugee* or survivor* or victim* or war).mp. [mp=title, book title, abstract, original title, name of substance word, subject heading word, floating sub-heading word, keyword heading word, organism supplementary concept word, protocol supplementary concept word, rare disease supplementary concept word, unique identifier, synonyms, population supplementary concept word, anatomy supplementary concept word] 341787

82 (stigma or help seeking).mp. [mp=title, book title, abstract, original title, name of substance word, subject heading word, floating sub-heading word, keyword heading word, organism supplementary concept word, protocol supplementary concept word, rare disease supplementary concept word, unique identifier, synonyms, population supplementary concept word, anatomy supplementary concept word] 45610

83 62 or 63 or 64 or 65 or 66 or 67 or 68 or 69 or 70 or 71 or 72 or 73 or 74 or 75 or 76 or 77 or 78 or 79 or 80 or 81 or 82 1965985

84 61 or 83 4851076

85 Randomized Controlled Trial.pt. 595975

86 controlled clinical trial.pt. 95361

87 randomi#ed.ab. 727388

88 randomly.ab. 411901

89 clinical trials as topic.sh. 201062

90 placebo.ab. 239664

91 trial.ti. 288529

92 85 or 86 or 87 or 88 or 89 or 90 or 91 1579559

93 exp animals/ not humans.sh. 5137154

94 92 not 93 1456057

95 Developing Countries/ 80967

96 (afghanistan or albania or algeria or angola or argentina or armenia or armenian or azerbaijan or bangladesh or republic of belarus or belarus or byelarus or belorussia or byelorussian or belize or benin or dahomey or bhutan or bolivia or "bosnia and herzegovina" or bosnia or herzegovina or botswana or bechuanaland or brazil or brasil or burkina faso or burkina fasso or upper volta or burundi or urundi or cabo verde or cape verde or cambodia or kampuchea or khmer republic or cameroon or cameron or cameroun or central african republic or ubangi shari or chad or china or colombia or comoros or comoro islands or iles comores or congo or zaire or costa rica or "cote d’ivoire" or "cote d’ ivoire" or cote divoire or cote d ivoire or ivory coast or cuba or djibouti or french somaliland or dominica or dominican republic or democratic republic of the congo or democratic republic congo or ecuador or egypt or united arab republic or el salvador or equatorial guinea or spanish guinea or eritrea or eswatini or swaziland or ethiopia or fiji or gabon or gabonese republic or gambia or georgia or georgian or ghana or gold coast or grenada or guatemala or guinea or guinea bissau or guyana or british guiana or haiti or hispaniola or honduras or hungary or india or indonesia or timor or iran or iraq or jamaica or jordan or kiribati or kazakhstan or kazakh or kenya or "democratic people’s republic of korea" or republic of korea or north korea or korea or kosovo or kyrgyzstan or kirghizia or kirgizstan or kyrgyz republic or kirghiz or laos or lao pdr or "lao people's democratic republic" or lebanon or lebanese republic or lesotho or basutoland or liberia or libya or libyan arab jamahiriya or republic of north macedonia or north macedonia or madagascar or malagasy republic or malawi or nyasaland or malaysia or malay federation or malaya federation or maldives or indian ocean islands or indian ocean or mali or micronesia or federated states of micronesia or marshall islands or nauru or northern mariana islands or palau or tuvalu or mauritania or mauritius or mexico or moldova or moldovian or mongolia or montenegro or montserrat or morocco or mozambique or myanmar or burma or namibia or nauru or nepal or nicaragua or niger or nigeria or niue or pakistan or panama or papua new guinea or new guinea or paraguay or peru or philippines or philipines or phillipines or phillippines or rwanda or ruanda or samoa or pacific islands or samoan islands or navigator island or navigator islands or "sao tome and principe" or senegal or serbia or sierra leone or solomon island or solomon islands or somalia or south africa or south sudan or sri lanka or ceylon or "saint helena" or "st helena" or saint lucia or "st lucia" or "saint vincent and the grenadines" or saint vincent or "st vincent" or grenadines or sudan or suriname or surinam or dutch guiana or netherlands guiana or syria or syrian arab republic or tajikistan or tadjikistan or tadzhikistan or tadzhik or tanzania or tanganyika or thailand or siam or timor leste or east timor or togo or togolese republic or tonga or tokelau or tunisia or turkey or turkmenistan or turkmen or tuvalu or uganda or ukraine or uzbekistan or uzbek or vanuatu or new hebrides or venezuela or vietnam or viet nam or middle east or west bank or gaza or "wallis and futuna" or wallis or futuna or yemen or yugoslavia or zambia or zimbabwe or northern rhodesia).mp. [mp=title, book title, abstract, original title, name of substance word, subject heading word, floating sub-heading word, keyword heading word, organism supplementary concept word, protocol supplementary concept word, rare disease supplementary concept word, unique identifier, synonyms, population supplementary concept word, anatomy supplementary concept word] 1991797

97 (global south or africa south of the sahara or sub-saharan africa or subsaharan africa or africa, central or central africa or africa, northern or north africa or northern africa or magreb or maghrib or sahara or africa, southern or southern africa or africa, eastern or east africa or eastern africa or africa, western or west africa or western africa or west indies or indian ocean islands or caribbean or central america or latin america or "south and central america" or south america or asia, central or central asia or asia, northern or north asia or northern asia or asia, southeastern or southeastern asia or south eastern asia or southeast asia or south east asia or asia, western or western asia or europe, eastern or east europe or eastern europe).mp. [mp=title, book title, abstract, original title, name of substance word, subject heading word, floating sub-heading word, keyword heading word, organism supplementary concept word, protocol supplementary concept word, rare disease supplementary concept word, unique identifier, synonyms, population supplementary concept word, anatomy supplementary concept word] 185093

98 (developing country or developing countries or developing nation? or developing population? or developing world or less developed countr* or less developed nation? or less developed population? or less developed world or lesser developed countr* or lesser developed nation? or lesser developed population? or lesser developed world or under developed countr* or under developed nation? or under developed population? or under developed world or underdeveloped countr* or underdeveloped nation? or underdeveloped population? or underdeveloped world or middle income countr* or middle income nation? or middle income population? or low income countr* or low income nation? or low income population? or lower income countr* or lower income nation? or lower income population? or underserved countr* or underserved nation? or underserved population? or underserved world or under served countr* or under served nation? or under served population? or under served world or deprived countr* or deprived nation? or deprived population? or deprived world or poor countr* or poor nation? or poor population? or poor world or poorer countr* or poorer nation? or poorer population? or poorer world or developing econom* or less developed econom* or lesser developed econom* or under developed econom* or underdeveloped econom* or middle income econom* or low income econom* or lower income econom* or low gdp or low gnp or low gross domestic or low gross national or lower gdp or lower gnp or lower gross domestic or lower gross national or lmic or lmics or third world or lami countr* or transitional countr* or emerging economies or emerging nation?).mp. [mp=title, book title, abstract, original title, name of substance word, subject heading word, floating sub-heading word, keyword heading word, organism supplementary concept word, protocol supplementary concept word, rare disease supplementary concept word, unique identifier, synonyms, population supplementary concept word, anatomy supplementary concept word] 199719

99 95 or 96 or 97 or 98 2162116

100 3 and 17 and 49 and 84 and 94 and 99 1411

101 limit 100 to (english language and yr="2018 -Current") 617

Embase <1974 to 2023 July 06>

1 juvenile/ or exp child/ or exp adolescent/ or young adult/ 4295594

2 (child* or boy* or girl* or kids or juvenil* or minors or paediatric* or pediatric* or adolesc* or preadolesc* or preadolesc* or pubert* or pubescen* or prepube* or prepube* or teen* or (young adj (adult* or people or patient* or men* or women* or male or female or survivor* or offender* or minorit*)) or youth* or student* or undergrad*).mp. [mp=title, abstract, heading word, drug trade name, original title, device manufacturer, drug manufacturer, device trade name, keyword heading word, floating subheading word, candidate term word] 5259508

3 1 or 2 5766593

4 school/ or college/ or community college/ or high school/ or kindergarten/ or middle school/ or nursery school/ or primary school/ or university/ 330642

5 education/ or curriculum/ or education program/ or learning environment/ or exp special education/ 606527

6 school health service/ 14876

7 exp student/ 318894

8 (preschool or nursery or kindergarten or school* or college* or campus* or classroom* or curricul* or teacher or gatekeeper or pupil*).mp. [mp=title, abstract, heading word, drug trade name, original title, device manufacturer, drug manufacturer, device trade name, keyword heading word, floating subheading word, candidate term word] 1855560

9 peer group/ 30203

10 ((peer or peers) adj (education or group or relation* or support* or intervention* or leader*)).mp. [mp=title, abstract, heading word, drug trade name, original title, device manufacturer, drug manufacturer, device trade name, keyword heading word, floating subheading word, candidate term word] 38177

11 student* union.mp. 65

12 ((primary or secondary or tertiary) adj educat*).mp. [mp=title, abstract, heading word, drug trade name, original title, device manufacturer, drug manufacturer, device trade name, keyword heading word, floating subheading word, candidate term word] 14567

13 ((church or communit* or holiday* or religi* or spiritual* or youth or vacation) adj2 (camp or club or group)).mp. [mp=title, abstract, heading word, drug trade name, original title, device manufacturer, drug manufacturer, device trade name, keyword heading word, floating subheading word, candidate term word] 7190

14 ((church or communit* or holiday* or religi* or spiritual* or youth or vacation) adj based).mp. [mp=title, abstract, heading word, drug trade name, original title, device manufacturer, drug manufacturer, device trade name, keyword heading word, floating subheading word, candidate term word] 101762

15 (university or universities).mp. [mp=title, abstract, heading word, drug trade name, original title, device manufacturer, drug manufacturer, device trade name, keyword heading word, floating subheading word, candidate term word] 887014

16 ((detention or refugee*) adj (camp* or centre* or center*)).mp. [mp=title, abstract, heading word, drug trade name, original title, device manufacturer, drug manufacturer, device trade name, keyword heading word, floating subheading word, candidate term word] 3259

17 4 or 5 or 6 or 7 or 8 or 9 or 10 or 11 or 12 or 13 or 14 or 15 or 16 3155746

18 mental health/ or community mental health/ or psychological well being/ 235384

19 mental stress/ or *stress/ 154049

20 *wellbeing/ 14899

21 (mental health or mental* ill* or psychiatric).mp. [mp=title, abstract, heading word, drug trade name, original title, device manufacturer, drug manufacturer, device trade name, keyword heading word, floating subheading word, candidate term word] 693484

22 (wellbeing or well being).mp. [mp=title, abstract, heading word, drug trade name, original title, device manufacturer, drug manufacturer, device trade name, keyword heading word, floating subheading word, candidate term word] 215211

23 (stress* or distress*).mp. [mp=title, abstract, heading word, drug trade name, original title, device manufacturer, drug manufacturer, device trade name, keyword heading word, floating subheading word, candidate term word] 1989939

24 18 or 19 or 20 or 21 or 22 or 23 2697534

25 depression/ or dysthymia/ or *major depression/ or "mixed anxiety and depression"/ 517426

26 mood disorder/ 52991

27 mood/ or *emotion/ 88020

28 (depress* or dysthymi* or affective disorder* or affective symptom* or mood* or mental).mp. [mp=title, abstract, heading word, drug trade name, original title, device manufacturer, drug manufacturer, device trade name, keyword heading word, floating subheading word, candidate term word] 1879888

29 ((Axis 1 or Axis I) adj disorder*).mp. [mp=title, abstract, heading word, drug trade name, original title, device manufacturer, drug manufacturer, device trade name, keyword heading word, floating subheading word, candidate term word] 2953

30 25 or 26 or 27 or 28 or 29 1905748

31 exp anxiety disorder/ 318430

32 *anxiety/ 66195

33 anxi*.mp. [mp=title, abstract, heading word, drug trade name, original title, device manufacturer, drug manufacturer, device trade name, keyword heading word, floating subheading word, candidate term word] 527483

34 (phobi* or agoraphobi* or PTSD or post trauma* or posttrauma or panic* or OCD or obsess* or compulsi* or GAD or stress disorder* or stress reaction* or acute stress or neurosis or neuroses or neurotic or psychoneuro* or (school adj2 (refusal or avoid*)) or social avoidance or mutism).mp. [mp=title, abstract, heading word, drug trade name, original title, device manufacturer, drug manufacturer, device trade name, keyword heading word, floating subheading word, candidate term word] 320334

35 (((anxi* or fear or fright) adj3 (perform* or athlet* or music* or act* or test* or exam*)) or math* anxiety).mp. [mp=title, abstract, heading word, drug trade name, original title, device manufacturer, drug manufacturer, device trade name, keyword heading word, floating subheading word, candidate term word] 33397

36 (public adj3 (speak* or speech)).mp. [mp=title, abstract, heading word, drug trade name, original title, device manufacturer, drug manufacturer, device trade name, keyword heading word, floating subheading word, candidate term word] 1830

37 31 or 32 or 33 or 34 or 35 or 36 799946

38 conduct disorder/ 8894

39 *behavior disorder/ 22966

40 psychosocial disorder/ 7648

41 juvenile delinquency/ or delinquency/ 11393

42 problem behavior/ 9339

43 *social adaptation/ 7425

44 ((behavi* or conduct or personalit*) adj2 (agressi* or nonagressi* or antisocial or anti social or dyssocial or defiant or delinquen* or disturb* or disrupt* or disorder* or internali#ing or externali#ing or problem*)).mp. [mp=title, abstract, heading word, drug trade name, original title, device manufacturer, drug manufacturer, device trade name, keyword heading word, floating subheading word, candidate term word] 192008

45 ((conduct or behavi* or antisocial or anti social or dyssocial or emotional* or internali#ing or externali#ing) adj3 (problem* of difficult* or psychopathol*)).mp. [mp=title, abstract, heading word, drug trade name, original title, device manufacturer, drug manufacturer, device trade name, keyword heading word, floating subheading word, candidate term word] 3381

46 (oppositional adj3 (defiant* or disorder*)).mp. [mp=title, abstract, heading word, drug trade name, original title, device manufacturer, drug manufacturer, device trade name, keyword heading word, floating subheading word, candidate term word] 5703

47 oppositional defiant disorder/ 3910

48 38 or 39 or 40 or 41 or 42 or 43 or 44 or 45 or 46 or 47 215868

49 24 or 30 or 37 or 48 4079161

50 primary prevention/ or secondary prevention/ 70785

51 Prevention/ or Preventive Medicine/ 337134

52 Prophylaxis/ 137547

53 health promotion/ or health education/ or health literacy/ 224834

54 prevent*.mp. [mp=title, abstract, heading word, drug trade name, original title, device manufacturer, drug manufacturer, device trade name, keyword heading word, floating subheading word, candidate term word] 3343338

55 ((early or brief) adj intervention*).mp. [mp=title, abstract, heading word, drug trade name, original title, device manufacturer, drug manufacturer, device trade name, keyword heading word, floating subheading word, candidate term word] 64193

56 ((universal or indicated or targeted) adj2 (program* or intervention*)).mp. [mp=title, abstract, heading word, drug trade name, original title, device manufacturer, drug manufacturer, device trade name, keyword heading word, floating subheading word, candidate term word] 38411

57 (vulnerabl* or at risk or (risk adj2 reduc*)).mp. [mp=title, abstract, heading word, drug trade name, original title, device manufacturer, drug manufacturer, device trade name, keyword heading word, floating subheading word, candidate term word] 784107

58 behavio* activation.mp. [mp=title, abstract, heading word, drug trade name, original title, device manufacturer, drug manufacturer, device trade name, keyword heading word, floating subheading word, candidate term word] 3154

59 (behavio* adj2 (intervention* or therap* or treatment* or psychotherap* or psychotherap*)).mp. [mp=title, abstract, heading word, drug trade name, original title, device manufacturer, drug manufacturer, device trade name, keyword heading word, floating subheading word, candidate term word] 123799

60 Behavior Therapy/ 45775

61 Cognitive Therapy/ 44380

62 self monitor*.mp. [mp=title, abstract, heading word, drug trade name, original title, device manufacturer, drug manufacturer, device trade name, keyword heading word, floating subheading word, candidate term word] 17791

63 (activit* adj3 (schedul* or plan* or arrang* or organis* or organiz*)).mp. [mp=title, abstract, heading word, drug trade name, original title, device manufacturer, drug manufacturer, device trade name, keyword heading word, floating subheading word, candidate term word] 33413

64 50 or 51 or 52 or 53 or 54 or 55 or 56 or 57 or 58 or 59 or 60 or 61 or 62 or 63 4317912

65 risk/ or risk factor/ 1768078

66 exp "accidents and accident related phenomena"/ 254520

67 exp emotional deprivation/ 60467

68 exp grief/ 15855

69 social problem/ or exp abuse/ or bullying/ or exp crime/ or divorce/ or exp human rights abuse/ or exp social discrimination/ or exp social exclusion/ or exp violence/ 484126

70 orphaned child/ 746

71 exp victim/ 32355

72 exp disaster/ 34543

73 life event/ 31971

74 coping behavior/ or runaway behavior/ 74459

75 "population and population related phenomena"/ or high risk population/ or minority group/ or rural population/ or urban population/ or vulnerable population/ 298198

76 exp survivor/ 109200

77 exp warfare/ 16332

78 conflict/ or family conflict/ 31470

79 early intervention/ 33536

80 (bereave* or bullying or divorce or foster care or grief or humanitarian or orphan* or RTA or refugee* or survivor* or victim* or war).mp. [mp=title, abstract, heading word, drug trade name, original title, device manufacturer, drug manufacturer, device trade name, keyword heading word, floating subheading word, candidate term word] 453238

81 (stigma or help seeking).mp. [mp=title, abstract, heading word, drug trade name, original title, device manufacturer, drug manufacturer, device trade name, keyword heading word, floating subheading word, candidate term word] 74258

82 65 or 66 or 67 or 68 or 69 or 70 or 71 or 72 or 73 or 74 or 75 or 76 or 77 or 78 or 79 or 80 or 81 3178325

83 64 or 82 6645212

84 randomized controlled trial/ 792300

85 (randomi#ed or randomi#ation).mp. [mp=title, abstract, heading word, drug trade name, original title, device manufacturer, drug manufacturer, device trade name, keyword heading word, floating subheading word, candidate term word] 1560204

86 (RCT or (random* adj3 (administ* or allocat* or assign* or class* or cluster* or control* or determine* or divide* or distribut* or expose* or fashion or number* or place* or recruit* or subsitut* or treat*))).ab. 897455

87 at random.ab. 16747

88 placebo.mp. [mp=title, abstract, heading word, drug trade name, original title, device manufacturer, drug manufacturer, device trade name, keyword heading word, floating subheading word, candidate term word] 524685

89 trial.ti,kw. 407480

90 (treatmentasusual or (treatment* adj2 usual) or (standard adj2 care) or (standard adj2 treatment) or (routine adj2 care) or (usual adj2 medication*) or (usual adj2 care) or TAU).mp. [mp=title, abstract, heading word, drug trade name, original title, device manufacturer, drug manufacturer, device trade name, keyword heading word, floating subheading word, candidate term word] 346937

91 (waitlist* or waitlist* or waitinglist* or wait* list* or (waiting adj (condition or control)) or WLC).mp. [mp=title, abstract, heading word, drug trade name, original title, device manufacturer, drug manufacturer, device trade name, keyword heading word, floating subheading word, candidate term word] 36658

92 (((delay* adj3 (start or treatment*)) or no intervention or no treatment* or notreatment or non treatment* or nontreatment* or nontreatment or minim* treatment* or untreated group* or untreated control* or without any treatment) and (control* or group*)).mp. [mp=title, abstract, heading word, drug trade name, original title, device manufacturer, drug manufacturer, device trade name, keyword heading word, floating subheading word, candidate term word] 122687

93 ((no intervention* or non intervention* or nonintervention* or without any intervention*) and (control* or group*)).mp. [mp=title, abstract, heading word, drug trade name, original title, device manufacturer, drug manufacturer, device trade name, keyword heading word, floating subheading word, candidate term word] 23796

94 (reference group or observation group or control group).mp. [mp=title, abstract, heading word, drug trade name, original title, device manufacturer, drug manufacturer, device trade name, keyword heading word, floating subheading word, candidate term word] 780403

95 84 or 85 or 86 or 87 or 88 or 89 or 90 or 91 or 92 or 93 or 94 3014621

96 Developing Country/ 101322

97 (afghanistan or albania or algeria or angola or argentina or armenia or armenian or azerbaijan or bangladesh or republic of belarus or belarus or byelarus or belorussia or byelorussian or belize or benin or dahomey or bhutan or bolivia or "bosnia and herzegovina" or bosnia or herzegovina or botswana or bechuanaland or brazil or brasil or burkina faso or burkina fasso or upper volta or burundi or urundi or cabo verde or cape verde or cambodia or kampuchea or khmer republic or cameroon or cameron or cameroun or central african republic or ubangi shari or chad or china or colombia or comoros or comoro islands or iles comores or congo or zaire or costa rica or "cote d’ivoire" or "cote d’ ivoire" or cote divoire or cote d ivoire or ivory coast or cuba or djibouti or french somaliland or dominica or dominican republic or democratic republic of the congo or democratic republic congo or ecuador or egypt or united arab republic or el salvador or equatorial guinea or spanish guinea or eritrea or eswatini or swaziland or ethiopia or fiji or gabon or gabonese republic or gambia or georgia or georgian or ghana or gold coast or grenada or guatemala or guinea or guinea bissau or guyana or british guiana or haiti or hispaniola or honduras or hungary or india or indonesia or timor or iran or iraq or jamaica or jordan or kiribati or kazakhstan or kazakh or kenya or "democratic people’s republic of korea" or republic of korea or north korea or korea or kosovo or kyrgyzstan or kirghizia or kirgizstan or kyrgyz republic or kirghiz or laos or lao pdr or "lao people's democratic republic" or lebanon or lebanese republic or lesotho or basutoland or liberia or libya or libyan arab jamahiriya or republic of north macedonia or north macedonia or madagascar or malagasy republic or malawi or nyasaland or malaysia or malay federation or malaya federation or maldives or indian ocean islands or indian ocean or mali or micronesia or federated states of micronesia or marshall islands or nauru or northern mariana islands or palau or tuvalu or mauritania or mauritius or mexico or moldova or moldovian or mongolia or montenegro or montserrat or morocco or mozambique or myanmar or burma or namibia or nauru or nepal or nicaragua or niger or nigeria or niue or pakistan or panama or papua new guinea or new guinea or paraguay or peru or philippines or philipines or phillipines or phillippines or rwanda or ruanda or samoa or pacific islands or samoan islands or navigator island or navigator islands or "sao tome and principe" or senegal or serbia or sierra leone or solomon island or solomon islands or somalia or south africa or south sudan or sri lanka or ceylon or "saint helena" or "st helena" or saint lucia or "st lucia" or "saint vincent and the grenadines" or saint vincent or "st vincent" or grenadines or sudan or suriname or surinam or dutch guiana or netherlands guiana or syria or syrian arab republic or tajikistan or tadjikistan or tadzhikistan or tadzhik or tanzania or tanganyika or thailand or siam or timor leste or east timor or togo or togolese republic or tonga or tokelau or tunisia or turkey or turkmenistan or turkmen or tuvalu or uganda or ukraine or uzbekistan or uzbek or vanuatu or new hebrides or venezuela or vietnam or viet nam or middle east or west bank or gaza or "wallis and futuna" or wallis or futuna or yemen or yugoslavia or zambia or zimbabwe or northern rhodesia).mp. [mp=title, abstract, heading word, drug trade name, original title, device manufacturer, drug manufacturer, device trade name, keyword heading word, floating subheading word, candidate term word] 2450175

98 (global south or africa south of the sahara or sub-saharan africa or subsaharan africa or africa, central or central africa or africa, northern or north africa or northern africa or magreb or maghrib or sahara or africa, southern or southern africa or africa, eastern or east africa or eastern africa or africa, western or west africa or western africa or west indies or indian ocean islands or caribbean or central america or latin america or "south and central america" or south america or asia, central or central asia or asia, northern or north asia or northern asia or asia, southeastern or southeastern asia or south eastern asia or southeast asia or south east asia or asia, western or western asia or europe, eastern or east europe or eastern europe).mp. [mp=title, abstract, heading word, drug trade name, original title, device manufacturer, drug manufacturer, device trade name, keyword heading word, floating subheading word, candidate term word] 205157

99 (developing country or developing countries or developing nation? or developing population? or developing world or less developed countr* or less developed nation? or less developed population? or less developed world or lesser developed countr* or lesser developed nation? or lesser developed population? or lesser developed world or under developed countr* or under developed nation? or under developed population? or under developed world or underdeveloped countr* or underdeveloped nation? or underdeveloped population? or underdeveloped world or middle income countr* or middle income nation? or middle income population? or low income countr* or low income nation? or low income population? or lower income countr* or lower income nation? or lower income population? or underserved countr* or underserved nation? or underserved population? or underserved world or under served countr* or under served nation? or under served population? or under served world or deprived countr* or deprived nation? or deprived population? or deprived world or poor countr* or poor nation? or poor population? or poor world or poorer countr* or poorer nation? or poorer population? or poorer world or developing econom* or less developed econom* or lesser developed econom* or under developed econom* or underdeveloped econom* or middle income econom* or low income econom* or lower income econom* or low gdp or low gnp or low gross domestic or low gross national or lower gdp or lower gnp or lower gross domestic or lower gross national or lmic or lmics or third world or lami countr* or transitional countr* or emerging economies or emerging nation?).mp. [mp=title, abstract, heading word, drug trade name, original title, device manufacturer, drug manufacturer, device trade name, keyword heading word, floating subheading word, candidate term word] 240751

100 96 or 97 or 98 or 99 2655672

101 3 and 17 and 49 and 83 and 95 and 100 2247

102 limit 101 to (english language and yr="2018 -Current") 1109

APA PsycInfo <1806 to June Week 4 2023>

1 pediatrics/ or child psychiatry/ or child psychopathology/ or child psychology/ or adolescent psychiatry/ or adolescent psychopathology/ or adolescent psychology/ or child psychotherapy/ or adolescent psychotherapy/ or childhood development/ or early childhood development/ or adolescent development/ 201731

2 (child* or boy* or girl* or kids or juvenil* or minors or paediatric* or pediatric* or adolesc* or preadolesc* or preadolesc* or pubert* or pubescen* or prepube* or prepube* or teen* or (young adj (adult* or people or patient* or men* or women* or male or female or survivor* or offender* or minorit*)) or youth* or student* or undergrad*).mp. [mp=title, abstract, heading word, table of contents, key concepts, original title, tests & measures, mesh word] 1914015

3 1 or 2 1914015

4 exp school based intervention/ 23400

5 education/ 44014

6 education/ or elementary education/ or high school education/ or higher education/ or middle school education/ or multicultural education/ or nontraditional education/ or preschool education/ or private school education/ or public school education/ or secondary education/ or special education/ 121700

7 schools/ or academic settings/ or boarding schools/ or charter schools/ or exp colleges/ or elementary schools/ or graduate schools/ or high schools/ or institutional schools/ or junior high schools/ or kindergartens/ or middle schools/ or nongraded schools/ or nursery schools/ 81023

8 school environment/ or college environment/ 17496

9 school facilities/ or campuses/ or classrooms/ or "learning centers (educational)"/ or school libraries/ 23598

10 community facilities/ or community mental health centers/ or exp libraries/ 5924

11 "summer camps (recreation)"/ 404

12 curriculum/ 29914

13 exp extracurricular activities/ or exp after school programs/ 3361

14 (preschool or nursery or kindergarten or school* or college* or campus* or classroom* or curricul* or teacher or gatekeeper or pupil*).mp. [mp=title, abstract, heading word, table of contents, key concepts, original title, tests & measures, mesh word] 967847

15 peers/ or peer counseling/ or peer tutoring/ 19848

16 ((peer or peers) adj (education or group or relation* or support* or intervention* or leader*)).mp. [mp=title, abstract, heading word, table of contents, key concepts, original title, tests & measures, mesh word] 40758

17 student* union.mp. 94

18 ((primary or secondary or tertiary) adj educat*).mp. [mp=title, abstract, heading word, table of contents, key concepts, original title, tests & measures, mesh word] 15453

19 ((church or communit* or holiday* or religi* or spiritual* or youth or vacation) adj2 (camp or club or group)).mp. [mp=title, abstract, heading word, table of contents, key concepts, original title, tests & measures, mesh word] 4883

20 ((church or communit* or holiday* or religi* or spiritual* or youth or vacation) adj based).mp. [mp=title, abstract, heading word, table of contents, key concepts, original title, tests & measures, mesh word] 38205

21 (university or universities).mp. [mp=title, abstract, heading word, table of contents, key concepts, original title, tests & measures, mesh word] 195670

22 ((detention or refugee*) adj (camp* or centre* or center*)).mp. [mp=title, abstract, heading word, table of contents, key concepts, original title, tests & measures, mesh word] 1700

23 4 or 5 or 6 or 7 or 8 or 9 or 10 or 11 or 12 or 13 or 14 or 15 or 16 or 17 or 18 or 19 or 20 or 21 or 22 1151145

24 mental health/ or well being/ 141438

25 Stress/ or Distress/ 102770

26 emotional adjustment/ 17220

27 "resilience (psychological)"/ or coping behavior/ or psychological stress/ 81914

28 (mental health or mental* ill* or psychiatric).mp. [mp=title, abstract, heading word, table of contents, key concepts, original title, tests & measures, mesh word] 549222

29 (wellbeing or well being).mp. [mp=title, abstract, heading word, table of contents, key concepts, original title, tests & measures, mesh word] 131706

30 (stress* or distress*).mp. [mp=title, abstract, heading word, table of contents, key concepts, original title, tests & measures, mesh word] 423181

31 24 or 25 or 26 or 27 or 28 or 29 or 30 996717

32 *affective disorders/ 12024

33 major depression/ or dysthymic disorder/ or reactive depression/ or "depression (emotion)"/ 176025

34 (depress* or dysthymi* or affective disorder* or affective symptom* or mood* or mental).mp. [mp=title, abstract, heading word, table of contents, key concepts, original title, tests & measures, mesh word] 1024130

35 ((Axis 1 or Axis I) adj disorder*).mp. [mp=title, abstract, heading word, table of contents, key concepts, original title, tests & measures, mesh word] 16399

36 32 or 33 or 34 or 35 1028685

37 exp anxiety/ 89724

38 anxiety disorders/ or acute stress disorder/ or death anxiety/ or generalized anxiety disorder/ or exp obsessive compulsive disorder/ or panic disorder/ or post-traumatic stress/ or exp posttraumatic stress disorder/ or separation anxiety disorder/ 88741

39 phobias/ or acrophobia/ or agoraphobia/ or claustrophobia/ or ophidiophobia/ or school phobia/ or social phobia/ 14293

40 fear/ or panic/ or panic attack/ 23151

41 anxi*.mp. [mp=title, abstract, heading word, table of contents, key concepts, original title, tests & measures, mesh word] 300594

42 (phobi* or agoraphobi* or PTSD or post trauma* or posttrauma or panic* or OCD or obsess* or compulsi* or GAD or stress disorder* or stress reaction* or acute stress or neurosis or neuroses or neurotic or psychoneuro* or (school adj2 (refusal or avoid*)) or social avoidance or mutism).mp. [mp=title, abstract, heading word, table of contents, key concepts, original title, tests & measures, mesh word] 200467

43 (((anxi* or fear or fright) adj3 (perform* or athlet* or music* or act* or test* or exam*)) or math* anxiety).mp. [mp=title, abstract, heading word, table of contents, key concepts, original title, tests & measures, mesh word] 24798

44 (public adj3 (speak* or speech)).mp. [mp=title, abstract, heading word, table of contents, key concepts, original title, tests & measures, mesh word] 2592

45 37 or 38 or 39 or 40 or 41 or 42 or 43 or 44 453454

46 conduct disorder/ or explosive disorder/ or oppositional defiant disorder/ 6136

47 *behavior disorders/ 7828

48 exp juvenile delinquency/ 20540

49 exp antisocial behavior/ 171722

50 ((behavi* or conduct or personalit*) adj2 (agressi* or nonagressi* or antisocial or anti social or dyssocial or defiant or delinquen* or disturb* or disrupt* or disorder* or internali#ing or externali#ing or problem*)).mp. [mp=title, abstract, heading word, table of contents, key concepts, original title, tests & measures, mesh word] 187935

51 ((conduct or behavi* or antisocial or anti social or dyssocial or emotional* or internali#ing or externali#ing) adj3 (problem* of difficult* or psychopathol*)).mp. [mp=title, abstract, heading word, table of contents, key concepts, original title, tests & measures, mesh word] 4017

52 (oppositional adj3 (defiant* or disorder*)).mp. [mp=title, abstract, heading word, table of contents, key concepts, original title, tests & measures, mesh word] 4476

53 46 or 47 or 48 or 49 or 50 or 51 or 52 337787

54 31 or 36 or 45 or 53 1797672

55 Preventive Mental Health Services/ or early intervention/ or "onset (disorders)"/ or health promotion/ or exp health education/ or health knowledge/ or health literacy/ or mental health programs/ or prevention/ or preventive medicine/ or public health/ 148305

56 prevent*.mp. [mp=title, abstract, heading word, table of contents, key concepts, original title, tests & measures, mesh word] 282019

57 ((early or brief) adj intervention*).mp. [mp=title, abstract, heading word, table of contents, key concepts, original title, tests & measures, mesh word] 28559

58 ((universal or indicated or targeted) adj2 (program* or intervention*)).mp. [mp=title, abstract, heading word, table of contents, key concepts, original title, tests & measures, mesh word] 12314

59 (vulnerabl* or at risk or (risk adj2 reduc*)).mp. [mp=title, abstract, heading word, table of contents, key concepts, original title, tests & measures, mesh word] 156408

60 behavio* activation.mp. [mp=title, abstract, heading word, table of contents, key concepts, original title, tests & measures, mesh word] 3340

61 (behavio* adj2 (intervention* or therap* or treatment* or psychotherap* or psychotherap*)).mp. [mp=title, abstract, heading word, table of contents, key concepts, original title, tests & measures, mesh word] 95875

62 Behavior Therapy/ 15981

63 Cognitive Therapy/ 14064

64 self monitor*.mp. [mp=title, abstract, heading word, table of contents, key concepts, original title, tests & measures, mesh word] 8882

65 (activit* adj3 (schedul* or plan* or arrang* or organis* or organiz*)).mp. [mp=title, abstract, heading word, table of contents, key concepts, original title, tests & measures, mesh word] 8765

66 55 or 56 or 57 or 58 or 59 or 60 or 61 or 62 or 63 or 64 or 65 605444

67 at risk populations/ or predisposition/ or risk factors/ or "susceptibility (disorders)"/ 146014

68 orphans/ or orphanages/ 1388

69 bullying/ or conflict/ or emotional abuse/ or school violence/ or teasing/ or threat/ or victimization/ 76114

70 school dropouts/ 0

71 runaway behavior/ 659

72 exp Crime Victims/ 5834

73 exp violent crime/ 47138

74 exp violence/ 123592

75 Trauma/ 29542

76 rural environments/ or urban environments/ or exp neighborhoods/ 55983

77 exp social issues/ 301211

78 war/ or conflict/ 39343

79 accidents/ or exp disasters/ or exp transportation accidents/ or Survivors/ 37106

80 bereavement/ or grief/ or divorce/ or child custody/ or parental death/ or exp parental absence/ 30624

81 life changes/ 5499

82 child abuse/ or abandonment/ or child neglect/ 35802

83 family conflict/ or domestic violence/ or emotional abuse/ 19581

84 (bereave* or bullying or divorce or foster care or grief or humanitarian or orphan* or RTA or refugee* or survivor* or victim* or war).mp. [mp=title, abstract, heading word, table of contents, key concepts, original title, tests & measures, mesh word] 222357

85 (stigma or help seeking).mp. [mp=title, abstract, heading word, table of contents, key concepts, original title, tests & measures, mesh word] 46602

86 67 or 68 or 69 or 70 or 71 or 72 or 73 or 74 or 75 or 76 or 77 or 78 or 79 or 80 or 81 or 82 or 83 or 84 or 85 745533

87 66 or 86 1179253

88 treatment effectiveness evaluation/ or clinical trials/ or mental health program evaluation/ or placebo/ 46514

89 (randomi#ed or randomi#ation).mp. [mp=title, abstract, heading word, table of contents, key concepts, original title, tests & measures, mesh word] 111615

90 (RCT or (random* adj3 (administ* or allocat* or assign* or class* or cluster* or control* or determine* or divide* or distribut* or expose* or fashion or number* or place* or recruit* or subsitut* or treat*))).ab. 116292

91 at random.ab. 3069

92 placebo.mp. [mp=title, abstract, heading word, table of contents, key concepts, original title, tests & measures, mesh word] 44261

93 trial.ti. 37787

94 (treatmentasusual or (treatment* adj2 usual) or (standard adj2 care) or (standard adj2 treatment) or (routine adj2 care) or (usual adj2 medication*) or (usual adj2 care) or TAU).mp. [mp=title, abstract, heading word, table of contents, key concepts, original title, tests & measures, mesh word] 35254

95 (waitlist* or waitlist* or waitinglist* or wait* list* or (waiting adj (condition or control)) or WLC).mp. [mp=title, abstract, heading word, table of contents, key concepts, original title, tests & measures, mesh word] 9720

96 (((delay* adj3 (start or treatment*)) or no intervention or no treatment* or notreatment or non treatment* or nontreatment* or nontreatment or minim* treatment* or untreated group* or untreated control* or without any treatment) and (control* or group*)).mp. [mp=title, abstract, heading word, table of contents, key concepts, original title, tests & measures, mesh word] 9681

97 ((no intervention* or non intervention* or nonintervention* or without any intervention*) and (control* or group*)).mp. [mp=title, abstract, heading word, table of contents, key concepts, original title, tests & measures, mesh word] 2739

98 (reference group or observation group or control group).mp. [mp=title, abstract, heading word, table of contents, key concepts, original title, tests & measures, mesh word] 84646

99 88 or 89 or 90 or 91 or 92 or 93 or 94 or 95 or 96 or 97 or 98 307098

100 Lower Income Level/ or Middle Income Level/ or Developing Countries/ 17737

101 (afghanistan or albania or algeria or angola or argentina or armenia or armenian or azerbaijan or bangladesh or republic of belarus or belarus or byelarus or belorussia or byelorussian or belize or benin or dahomey or bhutan or bolivia or "bosnia and herzegovina" or bosnia or herzegovina or botswana or bechuanaland or brazil or brasil or burkina faso or burkina fasso or upper volta or burundi or urundi or cabo verde or cape verde or cambodia or kampuchea or khmer republic or cameroon or cameron or cameroun or central african republic or ubangi shari or chad or china or colombia or comoros or comoro islands or iles comores or congo or zaire or costa rica or "cote d’ivoire" or "cote d’ ivoire" or cote divoire or cote d ivoire or ivory coast or cuba or djibouti or french somaliland or dominica or dominican republic or democratic republic of the congo or democratic republic congo or ecuador or egypt or united arab republic or el salvador or equatorial guinea or spanish guinea or eritrea or eswatini or swaziland or ethiopia or fiji or gabon or gabonese republic or gambia or georgia or georgian or ghana or gold coast or grenada or guatemala or guinea or guinea bissau or guyana or british guiana or haiti or hispaniola or honduras or hungary or india or indonesia or timor or iran or iraq or jamaica or jordan or kiribati or kazakhstan or kazakh or kenya or "democratic people’s republic of korea" or republic of korea or north korea or korea or kosovo or kyrgyzstan or kirghizia or kirgizstan or kyrgyz republic or kirghiz or laos or lao pdr or "lao people's democratic republic" or lebanon or lebanese republic or lesotho or basutoland or liberia or libya or libyan arab jamahiriya or republic of north macedonia or north macedonia or madagascar or malagasy republic or malawi or nyasaland or malaysia or malay federation or malaya federation or maldives or indian ocean islands or indian ocean or mali or micronesia or federated states of micronesia or marshall islands or nauru or northern mariana islands or palau or tuvalu or mauritania or mauritius or mexico or moldova or moldovian or mongolia or montenegro or montserrat or morocco or mozambique or myanmar or burma or namibia or nauru or nepal or nicaragua or niger or nigeria or niue or pakistan or panama or papua new guinea or new guinea or paraguay or peru or philippines or philipines or phillipines or phillippines or rwanda or ruanda or samoa or pacific islands or samoan islands or navigator island or navigator islands or "sao tome and principe" or senegal or serbia or sierra leone or solomon island or solomon islands or somalia or south africa or south sudan or sri lanka or ceylon or "saint helena" or "st helena" or saint lucia or "st lucia" or "saint vincent and the grenadines" or saint vincent or "st vincent" or grenadines or sudan or suriname or surinam or dutch guiana or netherlands guiana or syria or syrian arab republic or tajikistan or tadjikistan or tadzhikistan or tadzhik or tanzania or tanganyika or thailand or siam or timor leste or east timor or togo or togolese republic or tonga or tokelau or tunisia or turkey or turkmenistan or turkmen or tuvalu or uganda or ukraine or uzbekistan or uzbek or vanuatu or new hebrides or venezuela or vietnam or viet nam or middle east or west bank or gaza or "wallis and futuna" or wallis or futuna or yemen or yugoslavia or zambia or zimbabwe or northern rhodesia).mp. [mp=title, abstract, heading word, table of contents, key concepts, original title, tests & measures, mesh word] 262219

102 (global south or africa south of the sahara or sub-saharan africa or subsaharan africa or africa, central or central africa or africa, northern or north africa or northern africa or magreb or maghrib or sahara or africa, southern or southern africa or africa, eastern or east africa or eastern africa or africa, western or west africa or western africa or west indies or indian ocean islands or caribbean or central america or latin america or "south and central america" or south america or asia, central or central asia or asia, northern or north asia or northern asia or asia, southeastern or southeastern asia or south eastern asia or southeast asia or south east asia or asia, western or western asia or europe, eastern or east europe or eastern europe).mp. [mp=title, abstract, heading word, table of contents, key concepts, original title, tests & measures, mesh word] 24916

103 (developing country or developing countries or developing nation? or developing population? or developing world or less developed countr* or less developed nation? or less developed population? or less developed world or lesser developed countr* or lesser developed nation? or lesser developed population? or lesser developed world or under developed countr* or under developed nation? or under developed population? or under developed world or underdeveloped countr* or underdeveloped nation? or underdeveloped population? or underdeveloped world or middle income countr* or middle income nation? or middle income population? or low income countr* or low income nation? or low income population? or lower income countr* or lower income nation? or lower income population? or underserved countr* or underserved nation? or underserved population? or underserved world or under served countr* or under served nation? or under served population? or under served world or deprived countr* or deprived nation? or deprived population? or deprived world or poor countr* or poor nation? or poor population? or poor world or poorer countr* or poorer nation? or poorer population? or poorer world or developing econom* or less developed econom* or lesser developed econom* or under developed econom* or underdeveloped econom* or middle income econom* or low income econom* or lower income econom* or low gdp or low gnp or low gross domestic or low gross national or lower gdp or lower gnp or lower gross domestic or lower gross national or lmic or lmics or third world or lami countr* or transitional countr* or emerging economies or emerging nation?).mp. [mp=title, abstract, heading word, table of contents, key concepts, original title, tests & measures, mesh word] 29462

104 100 or 101 or 102 or 103 297709

105 3 and 23 and 54 and 87 and 99 and 104 796

106 limit 105 to (english language and yr="2018 -Current") 317

CENTRAL, 07/07/2023 18:13:58

#1 MeSH descriptor: [Child] explode all trees

#2 MeSH descriptor: [Adolescent] this term only

#3 MeSH descriptor: [Young Adult] this term only

#4 (child* or boy* or girl* or kids or juvenil* or minors or paediatric* or pediatric* or adolesc* or preadolesc* or pre-adolesc* or pubert* or pubescen* or prepube* or pre-pube* or teen* or (young next (adult* or people or patient* or men* or women* or male or female or survivor* or offender* or minorit*)) or youth* or student* or undergrad*):ti,ab,kw

#5 (#1 or #2 or #3 or #4 )

#6 MeSH descriptor: [Education] this term only

#7 MeSH descriptor: [Schools] this term only

#8 MeSH descriptor: [Schools, Nursery] this term only

#9 MeSH descriptor: [Students] this term only

#10 MeSH descriptor: [Universities] this term only

#11 (preschool or nursery or kindergarten or school* or college* or campus* or classroom* or curricul* or teacher or gatekeeper or pupil*):ti,ab,kw

#12 MeSH descriptor: [Peer Group] this term only

#13 ((peer or peers) next (education or group or relation* or support* or intervention* or leader*)):ti,ab,kw

#14 (student* next union):ti,ab,kw

#15 ((church or communit* or holiday* or religi* or spiritual* or youth or vacation) near/2 (camp or club or group)):ti,ab,kw

#16 ((church or communit* or holiday* or religi* or spiritual* or youth or vacation) next based):ti,ab,kw

#17 (university or universities):ti,ab,kw

#18 ((primary or secondary or tertiary) next educat*):ti,ab,kw

#19 ((detention or refugee*) next (camp* or centre* or center*)):ti,ab,kw

#20 #6 or #7 or #8 or #9 or #10 or #11 or #12 or #13 or #14 or #15 or #16 or #17 or #18 or #19

#21 MeSH descriptor: [Depression] this term only

#22 MeSH descriptor: [Depressive Disorder] this term only

#23 MeSH descriptor: [Mood Disorders] this term only

#24 (depress* or dysthymi* or affective disorder* or affective symptom* or mood* or mental):ti,ab,kw

#25 ((axis 1 or axis I) next disorder*):ti,ab,kw

#26 MeSH descriptor: [Anxiety Disorders] explode all trees

#27 MeSH descriptor: [Anxiety] this term only

#28 MeSH descriptor: [Performance Anxiety] this term only

#29 (anxi*):ti,ab,kw

#30 (phobi* or agoraphobi* or PTSD or post trauma* or posttrauma or panic* or OCD or obsess* or compulsi* or GAD or stress disorder* or stress reaction* or acute stress or neurosis or neuroses or neurotic or psychoneuro* or (school near/2 (refusal or avoid*)) or social avoidance or mutism):ti,ab,kw

#31 (((anxi* or fear or fright) near/3 (perform* or athlet* or music* or act* or test* or exam*)) or math* anxiety):ti,ab,kw

#32 (public near/3 (speak* or speech)):ti,ab,kw

#33 MeSH descriptor: [Conduct Disorder] this term only

#34 MeSH descriptor: [Child Behavior Disorders] this term only

#35 MeSH descriptor: [Juvenile Delinquency] this term only

#36 MeSH descriptor: [Social Behavior] this term only

#37 MeSH descriptor: [Social Behavior Disorders] explode all trees

#38 ((behavi* or conduct or personalit*) near/2 (agressi* or nonagressi* or antisocial or anti social or dyssocial or defiant or delinquen* or disturb* or disrupt* or disorder* or internalizing or externalizing or internalising or externalising or problem*)):ti,ab,kw

#39 ((conduct or behavi* or antisocial or anti social or dyssocial or emotional* or internalizing or externalizing or internalising or externalising) near/3 (problem* of difficult* or psychopathol*)):ti,ab,kw

#40 (oppositional near/3 (defiant* or disorder*)):ti,ab,kw

#41 MeSH descriptor: [Adaptation, Physiological] this term only

#42 MeSH descriptor: [Emotions] this term only

#43 MeSH descriptor: [Mental Health] this term only

#44 MeSH descriptor: [Social Adjustment] this term only

#45 MeSH descriptor: [Stress, Psychological] this term only

#46 ((mental health or mental* ill* or psychiatric)):ti,ab,kw

#47 ((wellbeing or well-being or "well being")):ti,ab,kw

#48 (stress* or distress*):ti,ab,kw

#49 #21 or #22 or #23 or #24 or #25 or #26 or #27 or #28 or #29 or #30 or #31 or #32 or #33 or #34 or #35 or #36 or #37 or #38 or #39 or #40 or #41 or #42 or #43 or #44 or #45 or #46 or #47 or #48

#50 MeSH descriptor: [Preventive Health Services] this term only

#51 MeSH descriptor: [Early Intervention, Educational] this term only

#52 MeSH descriptor: [Health Literacy] this term only

#53 MeSH descriptor: [Patient Education as Topic] this term only

#54 MeSH descriptor: [Health Promotion] this term only

#55 MeSH descriptor: [Primary Prevention] this term only

#56 MeSH descriptor: [Secondary Prevention] this term only

#57 (prevent*):ti,ab,kw

#58 ((early or brief) next intervention*):ti,ab,kw

#59 ((universal or indicated or targeted) near/2 (program* or intervention*)):ti,ab,kw

#60 (vulnerabl* or "at risk" or (risk near/2 reduc*)):ti,ab,kw

#61 MeSH descriptor: [Behavior Therapy] explode all trees

#62 MeSH descriptor: [Cognitive Behavioral Therapy] explode all trees

#63 (behavio* activation):ti,ab,kw

#64 (behavio* near/2 (intervention* or therap* or treatment* or psychotherap* or psychotherap*)):ti,ab,kw

#65 (self monitor*):ti,ab,kw

#66 (activit* near/3 (schedul* or plan* or arrang* or organis* or organiz*)):ti,ab,kw

#67 MeSH descriptor: [Risk] explode all trees

#68 MeSH descriptor: [Accidents] explode all trees

#69 MeSH descriptor: [Bereavement] explode all trees

#70 MeSH descriptor: [Bullying] this term only

#71 MeSH descriptor: [Child of Impaired Parents] this term only

#72 MeSH descriptor: [Child, Orphaned] this term only

#73 MeSH descriptor: [Crime Victims] this term only

#74 MeSH descriptor: [Disasters] explode all trees

#75 MeSH descriptor: [Divorce] explode all trees

#76 MeSH descriptor: [Life Change Events] this term only

#77 MeSH descriptor: [Runaway Behavior] this term only

#78 MeSH descriptor: [Urban Population] this term only

#79 MeSH descriptor: [Rural Population] this term only

#80 MeSH descriptor: [Survivors] this term only

#81 MeSH descriptor: [Violence] explode all trees

#82 MeSH descriptor: [Warfare] explode all trees

#83 MeSH descriptor: [Family Conflict] this term only

#84 MeSH descriptor: [Psychosocial Deprivation] this term only

#85 MeSH descriptor: [Poverty] this term only

#86 ((bereave* or bullying or divorce or foster care or grief or humanitarian or orphan* or RTA or refugee* or survivor* or victim* or war)):ti,ab,kw

#87 (stigma or help seeking):ti,ab,kw

#88 (#50 or #51 or #52 or #53 or #54 or #55 or #56 or #57 or #58 or #59 or #60 or #61 or #62 or #63 or #64 or #65 or #66 or #67 or #68 or #69 or #70 or #71 or #72 or #73 or #74 or #75 or #76 or #77 or #78 or #79 or #80 or #81 or #82 or #83 or #84 or #85 or #86 or #87)

#89 MeSH descriptor: [Developing Countries] this term only

#90 ((afghanistan OR albania OR algeria OR angola OR argentina OR armenia OR armenian OR azerbaijan OR bangladesh OR republic of belarus OR belarus OR byelarus OR belorussia OR byelorussian OR belize OR benin OR dahomey OR bhutan OR bolivia OR "bosnia and herzegovina" OR bosnia OR herzegovina OR botswana OR bechuanaland OR brazil OR brasil OR burkina faso OR burkina fasso OR upper volta OR burundi OR urundi OR cabo verde OR cape verde OR cambodia OR kampuchea OR khmer republic OR cameroon OR cameron OR cameroun OR central african republic OR ubangi shari OR chad OR china OR colombia OR comoros OR comoro islands OR iles comores OR congo OR zaire OR costa rica OR "cote d’ivoire" OR "cote d’ ivoire" OR cote divoire OR cote d ivoire OR ivory coast OR cuba OR djibouti OR french somaliland OR dominica OR dominican republic OR democratic republic of the congo OR democratic republic congo OR ecuador OR egypt OR united arab republic OR el salvador OR equatorial guinea OR spanish guinea OR eritrea OR eswatini OR swaziland OR ethiopia OR fiji OR gabon OR gabonese republic OR gambia OR georgia OR georgian OR ghana OR gold coast OR grenada OR guatemala OR guinea OR guinea bissau OR guyana OR british guiana OR haiti OR hispaniola OR honduras OR hungary OR india OR indonesia OR timor OR iran OR iraq OR jamaica OR jordan OR kiribati OR kazakhstan OR kazakh OR kenya OR "democratic people’s republic of korea" OR republic of korea OR north korea OR korea OR kosovo OR kyrgyzstan OR kirghizia OR kirgizstan OR kyrgyz republic OR kirghiz OR laos OR lao pdr OR "lao people's democratic republic" OR lebanon OR lebanese republic OR lesotho OR basutoland OR liberia OR libya OR libyan arab jamahiriya OR republic of north macedonia OR north macedonia OR madagascar OR malagasy republic OR malawi OR nyasaland OR malaysia OR malay federation OR malaya federation OR maldives OR indian ocean islands OR indian ocean OR mali OR micronesia OR federated states of micronesia OR marshall islands OR nauru OR northern mariana islands OR palau OR tuvalu OR mauritania OR mauritius OR mexico OR moldova OR moldovian OR mongolia OR montenegro OR montserrat OR morocco OR mozambique OR myanmar OR burma OR namibia OR nauru OR nepal OR nicaragua OR niger OR nigeria OR niue OR pakistan OR panama OR papua new guinea OR new guinea OR paraguay OR peru OR philippines OR philipines OR phillipines OR phillippines OR rwanda OR ruanda OR samoa OR pacific islands OR samoan islands OR navigator island OR navigator islands OR "sao tome and principe" OR senegal OR serbia OR sierra leone OR solomon island OR solomon islands OR somalia OR south africa OR south sudan OR sri lanka OR ceylon OR "saint helena" OR "st helena" OR saint lucia OR "st lucia" OR "saint vincent and the grenadines" OR saint vincent OR "st vincent" OR grenadines OR sudan OR suriname OR surinam OR dutch guiana OR netherlands guiana OR syria OR syrian arab republic OR tajikistan OR tadjikistan OR tadzhikistan OR tadzhik OR tanzania OR tanganyika OR thailand OR siam OR timor leste OR east timor OR togo OR togolese republic OR tonga OR tokelau OR tunisia OR turkey OR turkmenistan OR turkmen OR tuvalu OR uganda OR ukraine OR uzbekistan OR uzbek OR vanuatu OR new hebrides OR venezuela OR vietnam OR viet nam OR middle east OR west bank OR gaza OR "wallis and futuna" OR wallis OR futuna OR yemen OR yugoslavia OR zambia OR zimbabwe OR northern rhodesia)):ti,ab,kw

#91 ((global south OR africa south of the sahara OR sub-saharan africa OR subsaharan africa OR africa, central OR central africa OR africa, northern OR north africa OR northern africa OR magreb OR maghrib OR sahara OR africa, southern OR southern africa OR africa, eastern OR east africa OR eastern africa OR africa, western OR west africa OR western africa OR west indies OR indian ocean islands OR caribbean OR central america OR latin america OR "south and central america" OR south america OR asia, central OR central asia OR asia, northern OR north asia OR northern asia OR asia, southeastern OR southeastern asia OR south eastern asia OR southeast asia OR south east asia OR asia, western OR western asia OR europe, eastern OR east europe OR eastern europe)):ti,ab,kw

#92 ((developing country OR developing countries OR developing nation? OR developing population? OR developing world OR less developed countr* OR less developed nation? OR less developed population? OR less developed world OR lesser developed countr* OR lesser developed nation? OR lesser developed population? OR lesser developed world OR under developed countr* OR under developed nation? OR under developed population? OR under developed world OR underdeveloped countr* OR underdeveloped nation? OR underdeveloped population? OR underdeveloped world OR middle income countr* OR middle income nation? OR middle income population? OR low income countr* OR low income nation? OR low income population? OR lower income countr* OR lower income nation? OR lower income population? OR underserved countr* OR underserved nation? OR underserved population? OR underserved world OR under served countr* OR under served nation? OR under served population? OR under served world OR deprived countr* OR deprived nation? OR deprived population? OR deprived world OR poor countr* OR poor nation? OR poor population? OR poor world OR poorer countr* OR poorer nation? OR poorer population? OR poorer world OR developing econom* OR less developed econom* OR lesser developed econom* OR under developed econom* OR underdeveloped econom* OR middle income econom* OR low income econom* OR lower income econom* OR low gdp OR low gnp OR low gross domestic OR low gross national OR lower gdp OR lower gnp OR lower gross domestic OR lower gross national OR lmic OR lmics OR third world OR lami countr* OR transitional countr* OR emerging economies OR emerging nation?)):ti,ab,kw

#93 #89 or #90 or #91 or #92

#94 #5 and #20 and #49 and #88 and #93 with Publication Year from 2018 to 2023, in Trials

Filter: English language
