# Supplementary material for: School-based interventions to prevent anxiety and depression in children and adolescents in low- and middle-income countries: A systematic review
Source: PLoS One. 2025 Apr 30;20(4):e0316825. doi: 10.1371/journal.pone.0316825 (PMC12043155; doi:10.1371/journal.pone.0316825)
Supplement: S2 File — (DOCX) [file pone.0316825.s002.docx]

**Supplementary file 2**

## **Risk of bias assessments for included studies with justifications of ratings**

For individually randomised, parallel-group trials:

| **Study ID** | Osborn 2020 | | | **Aim** | assignment to intervention (the 'intention-to-treat' effect) |
| --- | --- | --- | --- | --- | --- |
| **Experimental** | Psychoeducation (Shamiri-Digital) | | | **Comparator** | Attention Control (Digital Study-skills) |
| **Outcome** | Anxiety and Depression Symptoms (GAD-7, PHQ-8) | | | **Results** | Depression: p=0.028, d=0.50, 95% CI (0.00, 1.60), Anxiety: p=0.280, d=0.29, 95% CI (0.20, 0.79) |
| **Domain** | **Signalling question** | | | **Response** | **Comments** |
| **Bias arising from the randomization process** | 1.1 Was the allocation sequence random? | | | Y | After recruitment, "...participants were randomly assigned to the intervention condition or study-skills condition using a random-number generator embedded in the study website. The study team was thus blind to this allocation" p.661 |
|  | 1.2 Was the allocation sequence concealed until participants were enrolled and assigned to interventions? | | | Y |  |
|  | 1.3 Did baseline differences between intervention groups suggest a problem with the randomization process? | | | PN | "There were more females (N=66, 64.08%) than males (N=37, 35.92%)" p.662.  "Correlations among baseline depressive and anxiety symptoms... were in the expected directions" p.662 These imbalances were likely compatible with chance, rather than a problem with randomisation. |
|  | **Risk of bias judgement** | | | **Low** |  |
| **Bias due to deviations from intended interventions** | 2.1.Were participants aware of their assigned intervention during the trial? | | | PN | "The study team... introduced and described the study as a program intended to improve wellness and acedemic functioning" p.660 "The study team was blinded to the allocation" p.661 Sufficient blinding of people delivering the interventions could be achieve. |
|  | 2.2.Were carers and people delivering the interventions aware of participants' assigned intervention during the trial? | | | N |  |
|  | 2.3. If Y/PY/NI to 2.1 or 2.2: Were there deviations from the intended intervention that arose because of the experimental context? | | | NA |  |
|  | 2.4 If Y/PY to 2.3: Were these deviations likely to have affected the outcome? | | | NA |  |
|  | 2.5. If Y/PY/NI to 2.4: Were these deviations from intended intervention balanced between groups? | | | NA |  |
|  | 2.6 Was an appropriate analysis used to estimate the effect of assignment to intervention? | | | Y | "...used an intent-to-treat approach and included all participants who had been randomised in our data analyses: p.661 |
|  | 2.7 If N/PN/NI to 2.6: Was there potential for a substantial impact (on the result) of the failure to analyse participants in the group to which they were randomized? | | | NA |  |
|  | **Risk of bias judgement** | | | **Low** |  |
| **Bias due to missing outcome data** | 3.1 Were data for this outcome available for all, or nearly all, participants randomized? | | | Y | Data were avaliable for 100% of those randomised. |
|  | 3.2 If N/PN/NI to 3.1: Is there evidence that result was not biased by missing outcome data? | | | NA |  |
|  | 3.3 If N/PN to 3.2: Could missingness in the outcome depend on its true value? | | | NA |  |
|  | 3.4 If Y/PY/NI to 3.3: Is it likely that missingness in the outcome depended on its true value? | | | NA |  |
|  | **Risk of bias judgement** | | | **Low** |  |
| **Bias in measurement of the outcome** | 4.1 Was the method of measuring the outcome inappropriate? | | | PN | "Two weeks after the intervention, participants completed study measures in their classrooms" p.661. Measures were self-reported. |
|  | 4.2 Could measurement or ascertainment of the outcome have differed between intervention groups? | | | N | Same measurement of outcome for both groups. |
|  | 4.3 Were outcome assessors aware of the intervention received by study participants? | | | PY | Whilst the study team was blinded to the allocation, information on outcomes were ascertained from participant self-report. Study participants might be aware of intervention status after the delivery of interventions. |
|  | 4.4 If Y/PY/NI to 4.3: Could assessment of the outcome have been influenced by knowledge of intervention received? | | | PY | Self-reported anxiety and depression outcome measure represents a participant-reported outcome. |
|  | 4.5 If Y/PY/NI to 4.4: Is it likely that assessment of the outcome was influenced by knowledge of intervention received? | | | PN |  |
|  | **Risk of bias judgement** | | | **Some concerns** |  |
| **Bias in selection of the reported result** | 5.1 Were the data that produced this result analysed in accordance with a pre-specified analysis plan that was finalized before unblinded outcome data were available for analysis? | | | NI | Referring to trail pre-registration at the Pan African Clinical Trials Registry (PACTR; registration number PACTR201906810558181), no specific information regarding pre-specified analysis plan was reported. |
|  | 5.2 ... multiple eligible outcome measurements (e.g. scales, definitions, time points) within the outcome domain? | | | N | All eligible outcome measurements within the outcome domain were reported. |
|  | 5.3 ... multiple eligible analyses of the data? | | | N | "Linear mixed models were used to compare intervention and control groups for each outcome measure" p.662. Eligible analyses of the data were reported. |
|  | **Risk of bias judgement** | | | **Some concerns** |  |
| **Overall bias** | **Risk of bias judgement** | | | **Some concerns** | Overall, this trial was rated as having some concerns for risk of bias. There was a lack of information on pre-specified analysis plan for which the results were produced. Also, there were some concerns with regards to the measurement of outcome which could be affected by participants' knowledge of intervention status. |
|  |  |  |  |  |  |
| **Study ID** | Rivero 2020 | | | **Aim** | assignment to intervention (the 'intention-to-treat' effect) |
| **Experimental** | CBT (FunFRIENDS program) | | | **Comparator** | Waiting List |
| **Outcome** | Anxiety Symptoms (PAS) | | | **Results** | ITT1: F=0.14, p=0.86, effect=0.00. ITT2: F=0.70, p=0.47, effect=0.01. |
| **Domain** | **Signalling question** | | | **Response** | **Comments** |
| **Bias arising from the randomization process** | 1.1 Was the allocation sequence random? | | | PY | "...children were randomly assigned to the Intervention Group (IG) or the Control Group (CG)" p.4499. There was no information reported about the sequence generation process or allocation sequence concealment. |
|  | 1.2 Was the allocation sequence concealed until participants were enrolled and assigned to interventions? | | | NI |  |
|  | 1.3 Did baseline differences between intervention groups suggest a problem with the randomization process? | | | N | "...21 children in the IG... and 22 children in the CG" p.4499. "ANOVA did not detect differences in age between participants in the IG and CG... no significant differences in the distribution of boys and girls in the two groups... did not detect differences between groups for any symptoms and behaviours analysed" p.4503 |
|  | **Risk of bias judgement** | | | **Some concerns** |  |
| **Bias due to deviations from intended interventions** | 2.1.Were participants aware of their assigned intervention during the trial? | | | PY | "During the intervention, the IG received a FunFRIENDS activity book... parents received a weekly report detailing the skills developed in each session" p.4501. Due to the nature/design of the intervention, sufficient blinding among participants, carers and trial personnel would not be achieved. |
|  | 2.2.Were carers and people delivering the interventions aware of participants' assigned intervention during the trial? | | | Y |  |
|  | 2.3. If Y/PY/NI to 2.1 or 2.2: Were there deviations from the intended intervention that arose because of the experimental context? | | | NI | There was no information reported regarding deviations. |
|  | 2.4 If Y/PY to 2.3: Were these deviations likely to have affected the outcome? | | | NA |  |
|  | 2.5. If Y/PY/NI to 2.4: Were these deviations from intended intervention balanced between groups? | | | NA |  |
|  | 2.6 Was an appropriate analysis used to estimate the effect of assignment to intervention? | | | Y | "...analyses were carried out using an Intention to Treat (ITT) model" p.4501 |
|  | 2.7 If N/PN/NI to 2.6: Was there potential for a substantial impact (on the result) of the failure to analyse participants in the group to which they were randomized? | | | NA |  |
|  | **Risk of bias judgement** | | | **Some concerns** |  |
| **Bias due to missing outcome data** | 3.1 Were data for this outcome available for all, or nearly all, participants randomized? | | | N | Data were available for 67% of those randomised, which is >5%. |
|  | 3.2 If N/PN/NI to 3.1: Is there evidence that result was not biased by missing outcome data? | | | PN | "...the last-observation-carried-forward (LOCF) procedure was used with those children who did not complete the intervention having their baseline values repeated at both T1 and T2... In the second protocol (ITT2), the mean values of the participants who completed the intervention at T1 and T2 from their respective groups were considered" p.4501. Although both analyses produce non-significant results, it is difficult to justify the missing outcome data was not biased as there was no specific analyses of the missing data. |
|  | 3.3 If N/PN to 3.2: Could missingness in the outcome depend on its true value? | | | PY | Missing data at T1: (n=2 IG, n=2 CG), T2: (n=5 IG, n=7 CG). |
|  | 3.4 If Y/PY/NI to 3.3: Is it likely that missingness in the outcome depended on its true value? | | | PN |  |
|  | **Risk of bias judgement** | | | **Some concerns** |  |
| **Bias in measurement of the outcome** | 4.1 Was the method of measuring the outcome inappropriate? | | | PN | "Parents/caregivers who signed... consent form and then completed the questionnaires" p.4501. Outcome measures were parent-reported. |
|  | 4.2 Could measurement or ascertainment of the outcome have differed between intervention groups? | | | N | Same measurement of outcome for both groups. |
|  | 4.3 Were outcome assessors aware of the intervention received by study participants? | | | Y | Parents were not blinded and were likely aware of the intervention received. |
|  | 4.4 If Y/PY/NI to 4.3: Could assessment of the outcome have been influenced by knowledge of intervention received? | | | PY | Parents' knowledge of intervention could affect the assessment of the outcome |
|  | 4.5 If Y/PY/NI to 4.4: Is it likely that assessment of the outcome was influenced by knowledge of intervention received? | | | PY |  |
|  | **Risk of bias judgement** | | | **High** |  |
| **Bias in selection of the reported result** | 5.1 Were the data that produced this result analysed in accordance with a pre-specified analysis plan that was finalized before unblinded outcome data were available for analysis? | | | NI | No information regarding pre-specified analysis plan was reported. |
|  | 5.2 ... multiple eligible outcome measurements (e.g. scales, definitions, time points) within the outcome domain? | | | N | All eligible outcome measurements within the outcome domain were reported. |
|  | 5.3 ... multiple eligible analyses of the data? | | | PN | "A chi-square test was used for analysis of categorical variables and a one-way analysis of variance for continuous variables. Two-way mixed ANOVA with repeated measures was used to evaluate the intervention... When significant effects were detected, Newman-Keuls post-hoc test was performed" p.4501. All eligible analyses of the data were reported. |
|  | **Risk of bias judgement** | | | **Some concerns** |  |
| **Overall bias** | **Risk of bias judgement** | | | **High** | Overall, this trial was rated as high risk of bias as participants, carers and trial personnel were not blinded to intervention allocation, influencing the measurement of outcomes. Additionally, there were some concerns regarding the absence of a pre-specified analysis plan before unblinded outcome data were available for analysis. There was also a lack of information regarding allocation sequence generation and concealment as well as missing outcome data. |

For cluster-randomised trials:

| **Study ID** | Ab Ghaffar 2019 | **Aim** | assignment to intervention (the 'intention-to-treat' effect) |
| --- | --- | --- | --- |
| **Experimental** | CBT (Information-Motivation-Behavioural-based program) | **Comparator** | Waiting List |
| **Outcome** | Anxiety Symptoms (RCADS) | **Results** | F(4,1097)=5.86, p=0.001, d=0.103 |
| **Domain** | **Signalling question** | **Response** | **Comments** |
| **Bias arising from the randomization process** | 1a.1 Was the allocation sequence random? | Y | "Once all the schools were enrolled, we randomly assigned schools (1:1) to be an intervention or control school. Randomisation was conducted at the school level in order to minimise possible contamination within schools. A simple randomisation protocol was used to randomise schools into the control and intervention groups. Firstly, number were assigned to the schools. The numbers were written on pieces of paper, folded and mixed up. The number were then picked at random. The first six primary schools were assigned as intervention schools and the remaining schools were assigned as control schools" p.4. There was no information about allocation concealment. |
|  | 1a.2 Was the allocation sequence concealed until clusters were enrolled and assigned to interventions? | NI |  |
|  | 1a.3 Did baseline differences between intervention groups suggest a problem with the randomization process? | N | "At baseline, there were no significant differences between the intervention and control groups for both categorical and continuous variables (p>0.05)" p.5 |
|  | **Risk of bias judgement** | **Some concerns** |  |
| **Bias arising from the timing of identification or recruitment of participants** | 1b.1 Were all the individual participants identified and recruited (if appropriate) before randomization of clusters? | **Y** | Schools and participants were enrolled (identified and recruited) before randomisation. |
|  | 1b.2 If N/PN/NI to 1b.1: Is it likely that selection of individual participants was affected by knowledge of the intervention assigned to the cluster? |  |  |
|  | 1b.3 Were there baseline imbalances that suggest differential identification or recruitment of individual participants between intervention groups? | **PN** | 5 schools assigned to intervention (n=193) and 6 schools assigned to control group (n=268). This imbalances in number of schools and participants recruited are unlikely to be due to differential identification or recruitment. |
|  | **Risk of bias judgement** | **Low** |  |
| **Bias due to deviations from intended interventions** | 2.1a Were participants aware that they were in a trial? | N | "Children were masked to the intervention allocation to maintain the blinding process throughout the study. The consent and information sheets for respondents and their parents or guardians only informed about the program in general; they did not contain specific information about the program they were going to receive - either intervention or control" p.4 |
|  | 2.1b If Y/PY/NI to 2.1a: Were participants aware of their assigned intervention during the trial? | NA | People delivering the interventions (research assistants) were not blinded. |
|  | 2.2 Were carers and people delivering the interventions aware of participants' assigned intervention during the trial? | Y |  |
|  | 2.3 If Y/PY/NI to 2.1b or 2.2: Were there deviations from the intended intervention that arose because of the trial context? | NI | There was no information about deviations reported. |
|  | 2.4 If Y/PY to 2.3: Were these deviations likely to have affected the outcome? | NA |  |
|  | 2.5 If Y/PY/NI to 2.4: Were these deviations from intended intervention balanced between groups? | NA |  |
|  | 2.6 Was an appropriate analysis used to estimate the effect of assignment to intervention? | Y | All participants allocated to intervention and control were included in analyses, suggesting an intention to treat approach - Figure 1 consort diagram. |
|  | 2.7 If N/PN/NI to 2.6: Was there potential for a substantial impact (on the result) of the failure to analyse participants in the group to which they were randomized? | NA |  |
|  | **Risk of bias judgement** | **Some concerns** |  |
| **Bias due to missing outcome data** | 3.1a Were data for this outcome available for all clusters that recruited participants? | Y | Data were available for all clusters. |
|  | 3.1b Were data for this outcome available for all, or nearly all, participants within clusters? | N | Data were available for 90% of those randomised. 10% loss to follow-up (>5%). |
|  | 3.2 If N/PN/NI to 3.1a or 3.1b: Is there evidence that the result was not biased by missing data? | PN | Analysis method did not correct for bias, nor were sensitivity analyses undertaken to investigate the potential effect of missing data. |
|  | 3.3 If N/PN to 3.2 Could missingness in the outcome depend on its true value? | PY | All missing data are due to loss to follow-up. 21 missing from intervention group and 27 missing from control group - Figure 1. |
|  | 3.4 If Y/PY/NI to 3.3: Is it likely that missingness in the outcome depended on its true value? | PN |  |
|  | **Risk of bias judgement** | **Some concerns** |  |
| **Bias in measurement of the outcome** | 4.1 Was the method of measuring the outcome inappropriate? | PN | "Child outcomes were collected during class time with self-completed questionnaires administered by research assistants at baseline, post-intervention and three months post-intervention" p.4. Outcome measures were self-reported. |
|  | 4.2 Could measurement or ascertainment of the outcome have differed between intervention groups? | N | Same methods utilised in both arms. |
|  | 4.3a If N/PN/NI to 4.1 and 4.2: Were outcome assessors aware that a trial was taking place? | N | Participants were blinded. |
|  | 4.3b If Y/PY/NI to 4.3a: Were outcome assessors aware of the intervention received by study participants? | NA |  |
|  | 4.4 If Y/PY/NI to 4.3b: Could assessment of the outcome have been influenced by knowledge of intervention received? | NA |  |
|  | 4.5 If Y/PY/NI to 4.4: Is it likely that assessment of the outcome was influenced by knowledge of intervention received? | NA |  |
|  | **Risk of bias judgement** | **Low** |  |
| **Bias in selection of the reported result** | 5.1 Were the data that produced this result analysed in accordance with a pre-specified analysis plan that was finalized before unblinded outcome data were available for analysis? | NI | No information about pre-specified analysis plan reported. |
|  | 5.2 ... multiple eligible outcome measurements (e.g. scales, definitions, time points) within the outcome domain? | N | All eligible outcome measurements within the outcome domain were reported. |
|  | 5.3 ... multiple eligible analyses of the data? | N | Eligible analyses of the data were reported. |
|  | **Risk of bias judgement** | **Some concerns** |  |
| **Overall bias** | **Risk of bias judgement** | **Some concerns** | Overall, this trial was rated as some concerns for risk of bias. There was no evidence of allocation concealment. Due to the design/nature of intervention, people delivering the interventions were not blinded which potentially could lead to deviations from intended intervention. Additionally, outcome data were missing for >5% of those randomised and analyses methods did not correct for bias nor were sensitivity analyses undertaken to investigate the potential effect of missing data. There was no evidence of a pre-specified analyses plan. |

| **Study ID** | Desan 2021 | | | **Aim** | assignment to intervention (the 'intention-to-treat' effect) |
| --- | --- | --- | --- | --- | --- |
| **Experimental** | Psychoeducation (Positive Psychology) | | | **Comparator** | Usual curriculum |
| **Outcome** | Depression Symptoms (CES-D) | | | **Results** | CES-D: Mean+/- SE=-0.725+/-0.436, 95% CI (-1.582, 0.1331), p>0.05 |
| **Domain** | **Signalling question** | | | **Response** | **Comments** |
| **Bias arising from the randomization process** | 1a.1 Was the allocation sequence random? | | | Y | "Classrooms were randomly assigned to intervention or control conditions by list alternation" p.1335. There was no information about allocation sequence concealment. |
|  | 1a.2 Was the allocation sequence concealed until clusters were enrolled and assigned to interventions? | | | NI |  |
|  | 1a.3 Did baseline differences between intervention groups suggest a problem with the randomization process? | | | PN | "The intervention and control groups did not differ significantly in age or gender... At the pre-semester point, the intervention group tended towards higher scores on measures of positive attitude and affect. The intervention group has a lower score on the CES-Depression measure (difference not significant)" p.1340. This imbalance is unlikely to be caused by problem with randomisation. |
|  | **Risk of bias judgement** | | | **Some concerns** |  |
| **Bias arising from the timing of identification or recruitment of participants** | 1b.1 Were all the individual participants identified and recruited (if appropriate) before randomization of clusters? | | | **PN** | "The study was presented to a parent meeting prior to the research. Five students were transferred from the experimental group, but the school policy is not to disclose whether these were due to withdrawal of consent or for other reason" p.1339. No further information reported to show the chronological order of identification, recruitment and randomisation process. |
|  | 1b.2 If N/PN/NI to 1b.1: Is it likely that selection of individual participants was affected by knowledge of the intervention assigned to the cluster? | | | **PN** | "Classrooms were randomly assigned to intervention or control conditions by list alternation" p.1335 |
|  | 1b.3 Were there baseline imbalances that suggest differential identification or recruitment of individual participants between intervention groups? | | | **PN** | “In the first cohort with 259 students, 127 students in five classrooms (taught by two teachers) received the intervention curriculum and 132 students in five classrooms (taught by one teacher) received the control curriculum (spring semester). In the second cohort with a separate group of 256 students, 125 students in four classrooms (taught by one teacher) received the intervention curriculum and 131 students in four classrooms (taught by one teacher) received the control curriculum (ensuing fall semester)” p.1335   There are imbalances in the number of participants in each intervention arms in both cohorts but there were equal allocations of classrooms. This is in keeping with a cluster RCT, unlikely to be due to differential identification or recruitment. Imbalances in characteristics were discussed in part 1a.3. |
|  | **Risk of bias judgement** | | | **Low** |  |
| **Bias due to deviations from intended interventions** | 2.1a Were participants aware that they were in a trial? | | | NI | "The study was presented to a parent meeting prior to the research" p.1339. There is a lack of information regarding what has been communicated and therefore, it is difficult to ascertain if participants would have known that they are in a trial. |
|  | 2.1b If Y/PY/NI to 2.1a: Were participants aware of their assigned intervention during the trial? | | | PY | Given the design/nature of intervention, people delivering the interventions (teachers) would be aware of the assigned intervention during the trials. Participants were also not blinded. |
|  | 2.2 Were carers and people delivering the interventions aware of participants' assigned intervention during the trial? | | | Y |  |
|  | 2.3 If Y/PY/NI to 2.1b or 2.2: Were there deviations from the intended intervention that arose because of the trial context? | | | NI | There was no information about deviations reported. |
|  | 2.4 If Y/PY to 2.3: Were these deviations likely to have affected the outcome? | | | NA |  |
|  | 2.5 If Y/PY/NI to 2.4: Were these deviations from intended intervention balanced between groups? | | | NA |  |
|  | 2.6 Was an appropriate analysis used to estimate the effect of assignment to intervention? | | | N | "A total of 580 students completed pre-semester questionnaires, but only 515 could be matched to post-semester questionnaires, largely due to errors in student identification number entry. Analysis was limited to the 515 participants with pre-post-semester data" p.1340. This reads as per-protocol analyses. |
|  | 2.7 If N/PN/NI to 2.6: Was there potential for a substantial impact (on the result) of the failure to analyse participants in the group to which they were randomized? | | | PY | >5% of participants were excluded from analysis due to error in student identification number entry |
|  | **Risk of bias judgement** | | | **High** |  |
| **Bias due to missing outcome data** | 3.1a Were data for this outcome available for all clusters that recruited participants? | | | Y | Data were available for all clusters |
|  | 3.1b Were data for this outcome available for all, or nearly all, participants within clusters? | | | Y | Of 515 included participants, data were available in 100% of participants. |
|  | 3.2 If N/PN/NI to 3.1a or 3.1b: Is there evidence that the result was not biased by missing data? | | | NA |  |
|  | 3.3 If N/PN to 3.2 Could missingness in the outcome depend on its true value? | | | NA |  |
|  | 3.4 If Y/PY/NI to 3.3: Is it likely that missingness in the outcome depended on its true value? | | | NA |  |
|  | **Risk of bias judgement** | | | **Low** |  |
| **Bias in measurement of the outcome** | 4.1 Was the method of measuring the outcome inappropriate? | | | PN | "Students completed identical pre- and post-semester questionnaires using an online system" p.1335. Outcome measures were self-reported. |
|  | 4.2 Could measurement or ascertainment of the outcome have differed between intervention groups? | | | N | Same methods utilised in both arms. |
|  | 4.3a If N/PN/NI to 4.1 and 4.2: Were outcome assessors aware that a trial was taking place? | | | PN | "Students were informed that all responses were analysed in the USA and would not be revealed to their teachers" p.1335. Outcome accessors (students) might be aware of receiving intervention but might not be aware that a trial was taking place. |
|  | 4.3b If Y/PY/NI to 4.3a: Were outcome assessors aware of the intervention received by study participants? | | | NA |  |
|  | 4.4 If Y/PY/NI to 4.3b: Could assessment of the outcome have been influenced by knowledge of intervention received? | | | NA |  |
|  | 4.5 If Y/PY/NI to 4.4: Is it likely that assessment of the outcome was influenced by knowledge of intervention received? | | | NA |  |
|  | **Risk of bias judgement** | | | **Low** |  |
| **Bias in selection of the reported result** | 5.1 Were the data that produced this result analysed in accordance with a pre-specified analysis plan that was finalized before unblinded outcome data were available for analysis? | | | NI | No information regarding pre-specified analysis plan finalised before unblinded outcome data were reported. |
|  | 5.2 ... multiple eligible outcome measurements (e.g. scales, definitions, time points) within the outcome domain? | | | N | All eligible outcome measurements within the outcome domain were reported. |
|  | 5.3 ... multiple eligible analyses of the data? | | | N | Eligible analyses of the data were reported. |
|  | **Risk of bias judgement** | | | **Some concerns** |  |
| **Overall bias** | **Risk of bias judgement** | | | **High** | Overall, this trial was rated as high risk of bias as there was a significant deviation from the intended interventions that arose from error in participants identification number entry resulting in participants who had been randomised to the intervention arms not being included in analyses. There was no prior trial protocol to determine whether the data that produced this result were analysed in accordance with a pre-specified analysis plan that was finalised before unblinded outcome data were available for analysis. There were some concerns with lack of information about allocation concealment. |
|  |  |  |  | |  |
| **Study ID** | Maalouf 2020 | | | **Aim** | assignment to intervention (the 'intention-to-treat' effect) |
| **Experimental** | CBT (My FRIENDS Youth program) | | | **Comparator** | Waiting List |
| **Outcome** | Anxiety and Depression Symptoms (SCARED and MFQ scales) | | | **Results** | Significant time*group effect for MFQ for depressive symptoms, p=0.039. Non-significant time*group effect for SCARED score, p=0.709. |
| **Domain** | **Signalling question** | | | **Response** | **Comments** |
| **Bias arising from the randomization process** | 1a.1 Was the allocation sequence random? | | | PY | "Schools with a sufficient number of consenting parents and assenting participants were randomised to either receive the intervention or control (n=5)" p.652. There was no further information about the process of allocation sequence randomisation or concealment. |
|  | 1a.2 Was the allocation sequence concealed until clusters were enrolled and assigned to interventions? | | | NI |  |
|  | 1a.3 Did baseline differences between intervention groups suggest a problem with the randomization process? | | | PY | "There were no between group differences in gender composition (p=0.279). Participants in the intervention group has a significantly higher frequency of unmarried biological/adoptive parents compared with control group (15% vs 5%; p=0.011). At baseline, participants in the intervention group have higher scores on...SCARED (p=0.002) and MFQ (p=0.001) than those in the control group (Table 1)." p.655. There is a uniform significant imbalance between the two arms, with intervention arms with higher levels of risks factors and depressive and anxiety scores, which could possibly suggest a problem with the randomisation process. |
|  | **Risk of bias judgement** | | | **High** |  |
| **Bias arising from the timing of identification or recruitment of participants** | 1b.1 Were all the individual participants identified and recruited (if appropriate) before randomization of clusters? | | | **Y** | "After a school agreed to participate… consent forms were sent out to parents along with parental data collection forms. After the baseline data collection, children of consenting parents who wished to participate in the study were assented. Schools with a sufficient number of consenting parents and assenting participants were then randomised to either intervention or control" p.652 |
|  | 1b.2 If N/PN/NI to 1b.1: Is it likely that selection of individual participants was affected by knowledge of the intervention assigned to the cluster? | | |  |  |
|  | 1b.3 Were there baseline imbalances that suggest differential identification or recruitment of individual participants between intervention groups? | | | **N** | There are imbalances in the number of participants in each arm but there was an equal allocation of schools. This is in keeping with a cluster RCT, unlikely to be due to differential identification or recruitment. Imbalances in characteristics were discussed in part 1a.3. |
|  | **Risk of bias judgement** | | | **Low** |  |
| **Bias due to deviations from intended interventions** | 2.1a Were participants aware that they were in a trial? | | | PN | Specific details of communication regarding the study were not reported. However, considering the study design with schools as unit of randomisation, participants would be aware of receiving an intervention but might not be aware that they were in a trial. |
|  | 2.1b If Y/PY/NI to 2.1a: Were participants aware of their assigned intervention during the trial? | | | NA | Given the design/nature of the intervention, people delivering the interventions (facilitators and co-facilitators) were aware of the assigned intervention during the trials. |
|  | 2.2 Were carers and people delivering the interventions aware of participants' assigned intervention during the trial? | | | Y |  |
|  | 2.3 If Y/PY/NI to 2.1b or 2.2: Were there deviations from the intended intervention that arose because of the trial context? | | | NI | There was no information about deviations available. |
|  | 2.4 If Y/PY to 2.3: Were these deviations likely to have affected the outcome? | | | NA |  |
|  | 2.5 If Y/PY/NI to 2.4: Were these deviations from intended intervention balanced between groups? | | | NA |  |
|  | 2.6 Was an appropriate analysis used to estimate the effect of assignment to intervention? | | | N | "Out of the 280 students enrolled... 277 provided baseline data and were included in the analysis" p.655, suggesting the use of per-protocol analysis. |
|  | 2.7 If N/PN/NI to 2.6: Was there potential for a substantial impact (on the result) of the failure to analyse participants in the group to which they were randomized? | | | PN | 3 students (n=1 intervention and n=2 control) were excluded due to their absence from school - Figure 1 CONSORT diagram. This is <5% of participants. |
|  | **Risk of bias judgement** | | | **Some concerns** |  |
| **Bias due to missing outcome data** | 3.1a Were data for this outcome available for all clusters that recruited participants? | | | Y | Data were available for all clusters. |
|  | 3.1b Were data for this outcome available for all, or nearly all, participants within clusters? | | | N | "Approximately 19% (n=52) of the 277 students who were included in the analysis did not have post-intervention data" p.655 |
|  | 3.2 If N/PN/NI to 3.1a or 3.1b: Is there evidence that the result was not biased by missing data? | | | Y | "...analysis was repeated after excluding participants who did not have post-intervention data (N=52), similar results regarding ... scores on the SCARED for anxiety symptoms... MFQ scores approached statistical significance (p=0.065)" p=655 |
|  | 3.3 If N/PN to 3.2 Could missingness in the outcome depend on its true value? | | | NA |  |
|  | 3.4 If Y/PY/NI to 3.3: Is it likely that missingness in the outcome depended on its true value? | | | NA |  |
|  | **Risk of bias judgement** | | | **Low** |  |
| **Bias in measurement of the outcome** | 4.1 Was the method of measuring the outcome inappropriate? | | | PN | "Participating students... asked to complete the self-report questionnaires" p.652. Outcome measures were self-reported. |
|  | 4.2 Could measurement or ascertainment of the outcome have differed between intervention groups? | | | N | Same methods utilised for both arms. |
|  | 4.3a If N/PN/NI to 4.1 and 4.2: Were outcome assessors aware that a trial was taking place? | | | PN | Participants were aware of receiving interventions/participating in a study but might not be aware of the trial. |
|  | 4.3b If Y/PY/NI to 4.3a: Were outcome assessors aware of the intervention received by study participants? | | | NA |  |
|  | 4.4 If Y/PY/NI to 4.3b: Could assessment of the outcome have been influenced by knowledge of intervention received? | | | NA |  |
|  | 4.5 If Y/PY/NI to 4.4: Is it likely that assessment of the outcome was influenced by knowledge of intervention received? | | | NA |  |
|  | **Risk of bias judgement** | | | **Low** |  |
| **Bias in selection of the reported result** | 5.1 Were the data that produced this result analysed in accordance with a pre-specified analysis plan that was finalized before unblinded outcome data were available for analysis? | | | NI | No information regarding pre-specified analysis plan was reported. |
|  | 5.2 ... multiple eligible outcome measurements (e.g. scales, definitions, time points) within the outcome domain? | | | N | All eligible outcome measurements within the outcome domain were reported. |
|  | 5.3 ... multiple eligible analyses of the data? | | | N | Eligible analyses of the data were reported. |
|  | **Risk of bias judgement** | | | **Some concerns** |  |
| **Overall bias** | **Risk of bias judgement** | | | **High** | Overall, this trial was rated as high risk of bias as there was a lack of information reported about randomisation/allocation methods as well as allocation concealment. Those assigned to the intervention arm had a significantly higher frequency of unmarried biological/adoptive parents and higher SCARED and MFQ scores than those in the control group, suggesting there may have been a problem with the randomisation process. Additionally, there were some concerns with deviation from intended intervention due to unblinded facilitators and inappropriate data analysis method. There was no prior trial protocol to determine whether the data that produced this result was analysed in accordance with a pre-specified analysis plan. |
|  |  |  |  | |  |
| **Study ID** | Singhal 2018 | | | **Aim** | assignment to intervention (the 'intention-to-treat' effect) |
| **Experimental** | CBT (Coping Skills program) | | | **Comparator** | Attention Control |
| **Outcome** | Depression Symptoms (CDI, CES-DC) | | | **Results** | CDI: F(1,90)=234.2, p<0.001 and CES-DC: F(1,90)=132.5, p<0.001 |
| **Domain** | **Signalling question** | | | **Response** | **Comments** |
| **Bias arising from the randomization process** | 1a.1 Was the allocation sequence random? | | | PY | "Schools were randomly assigned to intervention or control group to avoid contamination effects" p.100. There was no specific information reported regarding the allocation sequence generation process or concealment. |
|  | 1a.2 Was the allocation sequence concealed until clusters were enrolled and assigned to interventions? | | | NI |  |
|  | 1a.3 Did baseline differences between intervention groups suggest a problem with the randomization process? | | | N | "The intervention and control groups did not differ in their composition by grade, gender, birth order and age. They also did not differ by fathers' age, mothers' age. The intervention group has significantly more fathers educated up to Grade 12 and control group had significantly more fathers educated up to graduation. There was no difference between the intervention and control groups by family type and by parents' depressive symptom scores. Comparison between intervention and control groups at T1 indicated that the two groups did not differ significantly on each of the measures at baseline" p.100 |
|  | **Risk of bias judgement** | | | **Some concerns** |  |
| **Bias arising from the timing of identification or recruitment of participants** | 1b.1 Were all the individual participants identified and recruited (if appropriate) before randomization of clusters? | | | **Y** | Randomisation occurred after the identification of potential participants and recruitment of actual participants - Figure 1 |
|  | 1b.2 If N/PN/NI to 1b.1: Is it likely that selection of individual participants was affected by knowledge of the intervention assigned to the cluster? | | | **NA** |  |
|  | 1b.3 Were there baseline imbalances that suggest differential identification or recruitment of individual participants between intervention groups? | | | **N** | "One hundred and twenty students... comprised the intervention (n=65) and control (n=55) groups" p.100. Although there was imbalance in number of participants, but there was equal allocation of schools to each arm, in keeping with a cluster RCT. This cannot suggest differential identification or recruitment of participants. There were no imbalances in characteristics of study participants as discussed in part 1a.3. |
|  | **Risk of bias judgement** | | | **Low** |  |
| **Bias due to deviations from intended interventions** | 2.1a Were participants aware that they were in a trial? | | | PN | It is unclear what information was given to participants at recruitment. However, considering the nature/design of intervention which utilised schools as unit of randomisation, participants might be aware that they are receiving an intervention/participating in a study but not aware that they were in a trial. |
|  | 2.1b If Y/PY/NI to 2.1a: Were participants aware of their assigned intervention during the trial? | | | NA | Given that each school is assigned to either intervention or control groups, it is less likely that participants would be aware of their assigned intervention during the trial. People delivering (researchers) were aware of participants' assigned intervention during the trial. |
|  | 2.2 Were carers and people delivering the interventions aware of participants' assigned intervention during the trial? | | | Y |  |
|  | 2.3 If Y/PY/NI to 2.1b or 2.2: Were there deviations from the intended intervention that arose because of the trial context? | | | NI | There was no information about deviations reported. |
|  | 2.4 If Y/PY to 2.3: Were these deviations likely to have affected the outcome? | | | NA |  |
|  | 2.5 If Y/PY/NI to 2.4: Were these deviations from intended intervention balanced between groups? | | | NA |  |
|  | 2.6 Was an appropriate analysis used to estimate the effect of assignment to intervention? | | | N | Participants (n=14 intervention and n=6 control) were excluded from the analyses - Figure 1, suggesting a per protocol approach. No reasons identified for exclusion. |
|  | 2.7 If N/PN/NI to 2.6: Was there potential for a substantial impact (on the result) of the failure to analyse participants in the group to which they were randomized? | | | PY | >5% of participants were excluded from analyses in each arm. This could potentially have a substantial impact on the results. |
|  | **Risk of bias judgement** | | | **High** |  |
| **Bias due to missing outcome data** | 3.1a Were data for this outcome available for all clusters that recruited participants? | | | Y | Data were available for all clusters. |
|  | 3.1b Were data for this outcome available for all, or nearly all, participants within clusters? | | | NI | It is not clear how much data were available for all participants within clusters. |
|  | 3.2 If N/PN/NI to 3.1a or 3.1b: Is there evidence that the result was not biased by missing data? | | | N | Analysis method did not correct for bias, nor were sensitivity analyses undertaken to investigate the potential effect of missing data. |
|  | 3.3 If N/PN to 3.2 Could missingness in the outcome depend on its true value? | | | NI | No information about missingness of analysed data. Reasons for excluded data were also not reported. |
|  | 3.4 If Y/PY/NI to 3.3: Is it likely that missingness in the outcome depended on its true value? | | | NI |  |
|  | **Risk of bias judgement** | | | **High** |  |
| **Bias in measurement of the outcome** | 4.1 Was the method of measuring the outcome inappropriate? | | | PN | "All measures employed were self-report" p.103 |
|  | 4.2 Could measurement or ascertainment of the outcome have differed between intervention groups? | | | N | Same methods of measurements were utilised in both arms. |
|  | 4.3a If N/PN/NI to 4.1 and 4.2: Were outcome assessors aware that a trial was taking place? | | | PN | Considering the design with schools as unit of randomisation, participants would be aware of participating in interventions but might not be aware that a trial was taking place. |
|  | 4.3b If Y/PY/NI to 4.3a: Were outcome assessors aware of the intervention received by study participants? | | | NA |  |
|  | 4.4 If Y/PY/NI to 4.3b: Could assessment of the outcome have been influenced by knowledge of intervention received? | | | NA |  |
|  | 4.5 If Y/PY/NI to 4.4: Is it likely that assessment of the outcome was influenced by knowledge of intervention received? | | | NA |  |
|  | **Risk of bias judgement** | | | **Low** |  |
| **Bias in selection of the reported result** | 5.1 Were the data that produced this result analysed in accordance with a pre-specified analysis plan that was finalized before unblinded outcome data were available for analysis? | | | NI | No information about pre-specified analysis plan was reported. |
|  | 5.2 ... multiple eligible outcome measurements (e.g. scales, definitions, time points) within the outcome domain? | | | N | All eligible outcome measurements within the outcome domain were reported. |
|  | 5.3 ... multiple eligible analyses of the data? | | | N | Eligible analyses of the data were reported. |
|  | **Risk of bias judgement** | | | **Some concerns** |  |
| **Overall bias** | **Risk of bias judgement** | | | **High** | Overall, this trial was rated as high risk of bias as there was likely a significant deviation from the intended intervention which resulted in participants who has been randomised but failed to be included in analyses. Additionally, there were no information reported regarding missing data which could potentially impact the overall results. There were some concerns with having no pre-specified analysis plan and a lack of information regarding allocation sequence process and concealment. |
