# Supplementary material for: School-based interventions to prevent anxiety and depression in children and adolescents in low- and middle-income countries: A systematic review
Source: PLoS One. 2025 Apr 30;20(4):e0316825. doi: 10.1371/journal.pone.0316825 (PMC12043155; doi:10.1371/journal.pone.0316825)
Supplement: Attachment — Submitted filename: Response to Reviewers.docx [file pone.0316825.s007.docx]

Response to Reviewers comments:

We would like to thank the reviewers for their helpful comments. This letter includes the responds to each point raised by the academic editor and reviewer(s).

All line references are based on the document “Revised Manuscript with Tracked Changes”.

‘Response to reviewers’:

Reviewer 1

| 1. Method: Is there Prospero registration? If so, include the registration information and mention in the Method section. | The research protocol was retrospectively registered with INPLASY (<https://inplasy.com/projects/>), under registration number INPLASY2024100063. The registration information has been included to the Methods (Please see lines 146-147 ) and as one of the limitations in Discussion Sections (538-540): *‘This research was part of a master’s dissertation. Due to logistical challenges, including a tight timeframe and limited logistical support, the review protocol was not pre-registered at that time. However, it was subsequently registered retrospectively on INPLASY.’* |
| --- | --- |
| 2. In the narrative synthesis of the findings (270-370), study findings are discussed individually for each study. It would be helpful to also include a summary statement for each section (depression and anxiety, only depression and only anxiety), that brings together the findings. For instance, in the narrative synthesis for depression it would be helpful to summarise in a sentence, either at the beginning or at the end, that both studies found that the interventions led to a reduction in depressive symptoms, although for one of them the reduction did not reach a level of significance. | Thanks for your helpful suggestion. Summary statements have been included at related sections in Results: depression and anxiety, only depression and only anxiety (Please see lines 309-311, 345-347 & 379-380, also copy pasted here).  **depression and anxiety:**  *In summary, both studies demonstrated that school-based interventions led to a statistically significant reduction in depressive symptoms, while no significant effect was observed on anxiety symptoms.*  **only anxiety**  *In summary, both studies observed a reduction in anxiety symptoms. However, in contrast to the small but statistically significant reduction noted in the study by Ab Ghaffar et. al. (54), the results found by Rivero et al. (58) wereas not statistically significant***.**  **only depression**  *Both studies found that school-based interventions led to an improvement in depressive symptoms. However, only results found by Singhal et. al. (59) reached statistical significance.* |
| 3. The heterogeneity of outcome measures (450-451 and elsewhere in the text) especially in RCTs including minors has been noted in the literature before and highlighted as a problem in other systematic reviews – it would be helpful to highlight it further and cite some examples since it seems to be a big problem in being able to compare findings and ultimately address ‘what works for whom’ in terms of interventions. | Thanks for your reflection and suggestion.  This has been elaborated in the Discussion section, and several examples have been added (See lines 496-502 and related added references):  *‘Furthermore, the utilisation of a wide range of measurement tools across studies posed challenges in directly comparing the outcomes, impeding the synthesis of a cohesive overview. Our review highlights that the heterogeneity of outcome measures has posed a major challenge for comparing results and determining the effectiveness of interventions, aligning with previous literature (62,64,65). Standardising mental health outcome measures would facilitate comparisons across different approaches and programs in future studies (66).’*  We highlighted this further in the ‘Implications for Research and Practice’ section (Lines 561- 567):  *‘As mentioned before, the heterogeneity of outcome measures was highlighted as a significant issue in other systematic reviews that include RCTs on anxiety and depression in children and adolescents. This heterogeneity of outcome measures makes it difficult to compare findings across studies. Consequently, it becomes challenging to determine which interventions are effective for specific groups. Future trials should employ standardised measurement tools to enhance comparability and reliability.’* |
| 4. The review focuses on school-based interventions in low and middle-income countries, but the reviewed literature does not include any low-income countries since no studies were available for these. This finding deserves to be further highlighted in the discussion, not as a limitation of this review but as a worrisome finding for the lack of studies (and perhaps the lack of such interventions) in countries that may need it the most. | Thanks for your suggestion. This point has been highlighted in the discussion section, and its importance is explained accordingly (See lines 452-481).  Further emphasis has been included in the ‘Implications for Research and Practice’ section (See lines 557- 561):  *‘The current review emphasised the urgent need to design and implement more school-based preventive interventions for anxiety and depression, especially in LMICs where the need is the greatest.’* |
| 5. 488-491: It is unclear what is meant here by “the constraints imposed by the timeframe of this research” and how “given that studies with positive findings tend to have a higher likelihood of being published, this review is susceptible to publication bias.” Perhaps rephrase. Is the intended statement that the studies included in this review may have been susceptible to publication bias? | Many thanks for the suggestion to rephrase the sentence. Yes, the intended statement meant to convey that the studies included in this review may have been susceptible to publication bias.  This paragraph has been rephrased (See lines 539-547):  *‘This research was part of a master’s dissertation. Due to logistical challenges, including a tight timeframe and limited logistical support, the review protocol was not pre-registered at that time. However, it was subsequently registered retrospectively on INPLASY.*  *Due to the abovementioned challenges, tThe search was conducted in four electronic databases and included only the published articles. The exclusion of abstracts, ongoing trials without published outcomes and grey literature might result in the exclusion of valuable insights from unpublished research and studies with negative findings (67), which might lead to an incomplete and potentially skewed overview of the literature. Studies included in this review may have been susceptible to publication bias.’* |
| 6. Typos in need of correction:  - 149: “condition” instead of “conditions” - 155: “assessors” instead of “accessors” - 163: only English language studies as a significant limitation since many of the LMICs (and perhaps local research) may not be in English - 172: ideally include the precise date - 194: “assessors” instead of “accessors” - 276: “was observed” instead of “were observed” - 323: “that involved” instead of “that involving” - 368: “reduction” instead of “reductions” - 374: “were due” instead of “was due” - 394: “was deemed” instead of “were deemed” - 405: remove “exist” - 475: “reflects” instead of “reflects” - 476: “interventions” instead of “intervention” - 492: “the exclusion” instead of “the omission”? - 504: remove “a” from “a longer-term” - 516: remove “a” from “a more stringent inclusion criteria” - 522: “school-based measures” or “school-based prevention programs”? | Many thanks for identifying the Typos. Changes were made accordingly: line-   - 152: “condition” instead of “conditions” - 157, 196: “assessors” instead of “accessors” - 534-536: ‘Only English language studies were included’ -This has been recognized as a limitation in our review and mentioned in The Limitation part of the Discussion section. - 174: precise date was included - 204: addded- “assessors” instead of “accessors” - 279: addded- “was observed” instead of “were observed” - 329: addded- “that involved” instead of “that involving” - 377: addded- “reduction” instead of “reductions” - 385: addded- “were due” instead of “was due” - 405: addded- “was deemed” instead of “were deemed” - 416: removed- “exist”, and “to” changed to “that” - 526: addded- “reflect” instead of “reflects” - “intervention” – section deleted - 545: we added-“the exclusion” instead of “the omission” - 569: removed- “a” from “a longer-term” - 582: removed- “a” from “a more stringent inclusion criteria” - 588: “school-based measures” changed to “school-based prevention programs” |

Reviewer 2

| *I agree that many, if not all, of the systematic reviews are done on studies from Western countries with sound economic income. Studies of anxiety and stress prevention in children and adolescents in countries outside these criteria are scarce, as this study has shown. Suppose the reason they have chosen such studies in countries with middle or low-income levels is that it is assumed that there is a greater likelihood of pre-anxiety or pre-depressive symptoms simply because they have fewer resources. However, this assumption may be erroneous. The factors leading to these symptoms may differ between developed and developing countries. Social pressure is more significant in developed countries, and the protective role and values associated with the family may be higher in developing countries such as Brazil or China. | Many thanks for your sharing your reflection and insights on this. The aim of the review is to identify effective interventions for preventing anxiety and depression in children within school settings in LMICs, where research has indicated higher rates of these symptoms, as highlighted in our introduction. We are not suggesting that children in these regions have a greater likelihood of pre-anxiety or pre-depressive symptoms simply because of fewer resources. We fully agree that the factors contributing to these symptoms may differ between developed and developing countries, which underscores the need to expand research that distinguishes LMICs from HICs due to their unique contextual differences. As you pointed out, countries like Brazil and China may have protective family values, so considering cultural differences is another key reason for focusing more research on LMICs. These points has now been discussed in the Discussion section (Please see lines 444-484) |
| --- | --- |
| 1. Table 2 points out the cut-off point of anxiety and/or depression that marks the measure used to understand the previous levels better. | Many thanks for the suggestion. Cut-off scores have been included in Table 2 and additional information about cut-off scores have been added. |
| 2. Does this review require a code of Prospero or similar? This condition is usually necessary before its acceptance. | The research protocol was retrospectively registered with INPLASY (<https://inplasy.com/projects/>), under registration number INPLASY2024100063. The registration information has been included to the Methods (Please see lines 143-147 ) and as one of the limitations in Discussion Sections (lines 538-540): *‘This research was part of a master’s dissertation. Due to logistical challenges, including a tight timeframe and limited logistical support, the review protocol was not pre-registered at that time. However, it was subsequently registered retrospectively on INPLASY.’* |
| 3. To justify the inclusion of these countries, LMICs should have gone more profound in the discussion section of the similarities and differences with other reviews that would have included developed Western countries. They may share explanatory mechanisms and differences in different aspects may also be observed. | Thanks for your suggestion. Additional information has been included in the discussion section to justify the inclusion of these countries The similarities and differences with other reviews that included developed Western countries has been discussed within the scope of the review. (Please see lines 444-484) |

Other changes, including changes to reference list

| Line 151: “Studies included participants within the age range of 4 to 18 years at recruitment…” | Rephrased to “Studies with participants aged between 4 and 18 years at recruitment…” |
| --- | --- |
| Line 236: “All studies were conducted in middle-income countries…” | Abbreviations included in “All studies were conducted in middle-income countries (MICs)…” and utilised throughout the article. |
| Line 297: “In conclusion, my FRIENDS Youth program was effective in improving the depressive symptoms in….” | Deleted “In conclusion,” to improve the flow of the sentences. |
| Line 705: “This systematic review is a pioneering effort in focusing on school-based intervention to prevent anxiety and depression in children and young people within LMICs. A notable asset lies in its emphasis on public and stakeholder involvements. This engagement ensures that the research is informed by real-world perspectives, enhancing the relevance and applicability of the findings for the intended audience.” | Deleted this section regarding public and stakeholder involvements. |
| Lines 951-972: New references added to the reference list | Reference list has been updated to encompass new sources for the discussion section. |
